# Supplementary figures and images for: Impact of postpartum tenofovir-based antiretroviral therapy on bone mineral density in breastfeeding women with HIV enrolled in a randomized clinical trial
Source: PLoS One. 2021 Feb 5;16(2):e0246272. doi: 10.1371/journal.pone.0246272 (PMC7864465; doi:10.1371/journal.pone.0246272)

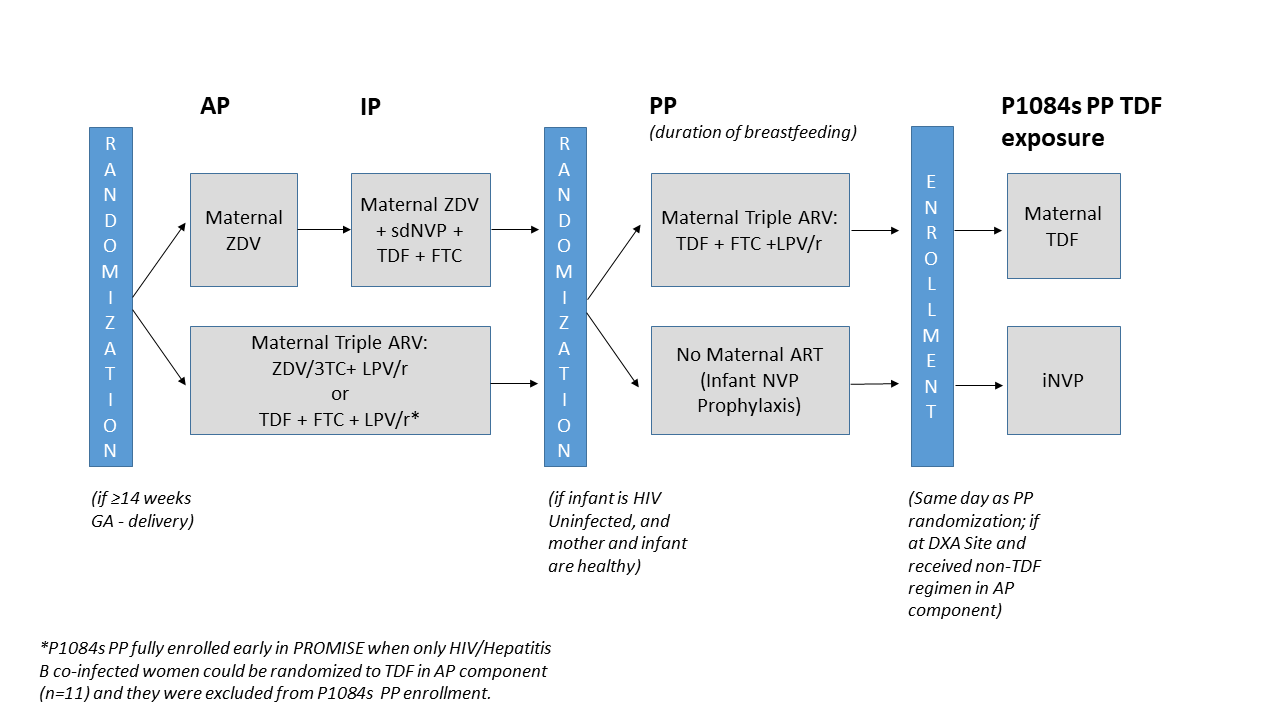

Supplement: S1 Fig — Under version 2.0 of the trial protocol only women who were positive for hepatitis B surface antigen were randomly assigned to tenofovir-based antiretroviral therapy (ART); under (last) version 3.0, all women could be assigned to any of the three regimens (1). P1084s postpartum enrolled completely under PROMISE protocol version 2.0. After delivery, women were randomized to continue or initiate ART or to no ART (2). In a third randomization (not shown) after weaning, eligible women were randomized again to continue ART or to a no ART arm (3). Those who had been previously randomized to no ART were continued on no ART. The first antepartum randomization was Apr. 11, 2011. After the results of the START study in July 2015 (4), all women were recommended to initiate ART. At that time, all P1084s women who enrolled in the Postpartum Exposure Component had discontinued or completed the 74 week follow-up for this substudy. All women who met specific treatment guidelines and all infants who were confirmed to be infected with the human immunodeficiency virus (HIV) began ART immediately. The above schema represents breast-feeding women. AP = Antepartum, IP = intrapartum, PP = postpartum. TDF = tenofovir disoproxil fumarate, FTC = emtricitabine, ZDV = zidovudine, sdNVP = single dose nevirapine. (TIF) [file pone.0246272.s001.tif]
