# Supplementary material for: Impact of postpartum tenofovir-based antiretroviral therapy on bone mineral density in breastfeeding women with HIV enrolled in a randomized clinical trial
Source: PLoS One. 2021 Feb 5;16(2):e0246272. doi: 10.1371/journal.pone.0246272 (PMC7864465; doi:10.1371/journal.pone.0246272)
Supplement: S1 Table — (DOCX) [file pone.0246272.s002.docx]

**S1 Table: Baseline Characteristics by P1084s Co-enrollment**

|  | | **Potentially Eligible for P1084s** | |  | |
| --- | --- | --- | --- | --- | --- |
|  | | **Enrolled (N=400)** | **Did Not Enroll (N=192)** | **Total (N=592)** | **P-Value** |
| Age (years) | Median (Q1-Q3) | 26.5 (23.3-30.2) | 27.5 (24.8-30.8) | 26.9 (23.8-30.5) | 0.03^a^ |
| Country | Malawi | 28 (7%) | 69 (36%) | 97 (16%) | <.001^b^ |
|  | South Africa | 38 (10%) | 63 (33%) | 101 (17%) |  |
|  | Uganda | 167 (42%) | 8 (4%) | 175 (30%) |  |
|  | Zimbabwe | 167 (42%) | 52 (27%) | 219 (37%) |  |
| Previous PROMISE Component | 1077BA – breastfeeding | 362 (91%) | 166 (86%) | 528 (89%) | 0.11^b^ |
|  | 1077BL – late presenters | 37 (9%) | 23 (12%) | 60 (10%) |  |
|  | 1077FA – formula feeding | 1 (0%) | 3 (2%) | 4 (1%) |  |
| PROMISE Antepartum Randomization | # missing (late presenters) | 37 | 23 | 60 |  |
|  | Triple ARV (3TC-ZDV/LPV-RTV) | 186 (51%) | 91 (54%) | 277 (52%) | 0.58^b^ |
|  | ZDV+sdNVP+TRV tail | 177 (49%) | 78 (46%) | 255 (48%) |  |
| Weight (kg) | Median (Q1-Q3) | 61.1 (55.7-70.0) | 62.5 (55.6-72.1) | 61.7 (55.6-70.8) | 0.31^a^ |
| Height (cm) | Median (Q1-Q3) | 159.0 (152.5-163.0) | 158.1 (154.9-162.0) | 159.0 (153.0-162.5) | 0.84^a^ |
| BMI (kg/m^2^) | Median (Q1-Q3) | 24.8 (22.4-28.0) | 24.9 (22.9-27.9) | 24.8 (22.6-27.9) | 0.53^a^ |
| CD4 at Antepartum Screening (cells/mm^3^) | # missing | 37 | 23 | 60 |  |
|  | Median (Q1-Q3) | 540 (446-702) | 521 (431-642) | 531.5 (439.5-677.0) | 0.12^a^ |
| CD4 at Postpartum Screening (cells/mm^3^)^c^ | # missing | 1 | 0 | 1 |  |
|  | Median (Q1-Q3) | 668 (544-855) | 644.0 (527.5-809.0) | 663 (541-842) | 0.31^a^ |
| HIV RNA level prior to randomization (copies/ml)^d^ | Median (Q1-Q3) | 400 (83-2222) | 400.0 (40.0-1442.5) | 400 (54-1922) | 0.02^a^ |
| WHO Stage^c^ | # missing | 338 | 158 | 496 |  |
|  | Clinical stage I | 57 (92%) | 32 (94%) | 89 (93%) | 0.25 |
| Number of years of smoking^c^ | # missing | 280 | 90 | 370 |  |
|  | No history of smoking | 119 (99%) | 102 (100%) | 221 (100%) |  |
| History of alcohol use prior to randomization^c^ | # missing | 324 | 159 | 483 |  |
|  | Yes | 9 (12%) | 1 (3%) | 10 (9%) | 0.14^b^ |
|  | | | | | |

^a^Wilcoxon Test; ^b^Chi-Square Test; ^c^Characteristics with small sample sizes (for WHO Stage, current and history of smoking, and alcohol use) may have had an evaluation >30 days before randomization. ^d^Assay lower limits of quantification differed by country and ranged from 20 to 400 copies/ml.  Adjusting for country, HIV RNA level p-value=0.40 (t-test).
