# Supplementary material for: Impact of postpartum tenofovir-based antiretroviral therapy on bone mineral density in breastfeeding women with HIV enrolled in a randomized clinical trial
Source: PLoS One. 2021 Feb 5;16(2):e0246272. doi: 10.1371/journal.pone.0246272 (PMC7864465; doi:10.1371/journal.pone.0246272)
Supplement: S1 File — (PDF) [file pone.0246272.s003.pdf]

IMPAACT P1084s

(DAIDS Document ID 10790)

Maternal and Infant Monitoring for Evidence of Toxicity Related to Tenofovir Exposure: The Bone and Kidney Health Substudy of the IMPAACT 1077 PROMISE Protocol (Promoting Maternal and Infant Survival Everywhere)

A Multicenter, International Trial of the
International Maternal Pediatric Adolescent AIDS
Clinical Trials Group (IMPAACT)

Sponsored by:

The National Institute of Allergy and Infectious Diseases (NIAID)
and
The *Eunice Kennedy Shriver*
National Institute of Child Health and Human Development (NICHD)

Pharmaceutical Support Provided by:

Gilead Sciences, Inc.

IND # 107,507

**The IMPAACT HIV/ARV Complications Committee Chair and Co-Chairs:**	Myron Levin, MD<br>William Borkowsky, MD<br>Werner Schimana, MD
**Protocol Co-Chair:**	George K. Siberry, MD, MPH
**Protocol Co-Chair:**	Lynda Stranix-Chibanda, MD
**NIAID Medical Officers and Medical Monitor**:	Lawrence Fox, MD, PhD<br>Renee Browning, RN, MSN
**NICHD Medical Officer:**	Lynne Mofenson, MD

Version 2.0
11 October 2012

Protocol Co-Chairs
George K. Siberry, MD, MPH
Pediatric, Adolescent and Maternal AIDS Branch
National Institute of Child Health and Human Development (NICHD)
US National Institutes of Health
Bethesda, MD 20892-7510
Phone: (301) 496-7350
Email: siberryg@mail.nih.gov

Lynda Stranix-Chibanda, MBChB, MMED
Department of Paediatrics and Child Health
College of Health Sciences
University of Zimbabwe,
P.O. Box A178, Avondale
Harare, Zimbabwe
Phone: +263 4 704890
Email: lynda@uz-ucsf.co.zw

PMTCT Chair
Mary Glenn Fowler, MD, MPH
Visiting Professor, Department of Pathology
Johns Hopkins Medical Institute
Makerere University (MU)-Johns Hopkins University (JHU) Research Collaboration
P.O. Box 23491
Kampala, Uganda
Phone: +256 414 532 091
Email: mgfowler@mujhu.org

PMTCT Vice Chair
Tsungai Chipato, MBChB, FRCOG, MCE
Department of Obstetrics and Gynaecology
College of Health Sciences
University of Zimbabwe,
P.O. Box A178, Avondale
Harare, Zimbabwe
Phone: +263 4 308848
Email: tchipato@zol.co.zw

Maternal Health Chair
Judith Currier, MD, MSc
Professor of Medicine
UCLA CARE Center
9911 W Pico Boulevard, Suite 980
Los Angeles, CA 90035
Phone: (310) 557-1891
Email: jscurrier@mednet.ucla.edu

NIAID Medical Officers
Lawrence Fox, MD, Ph.D.
TRP, DAIDS, NIAID, NIH
6700-B Rockledge Drive, MSC 7624
Bethesda, MD 20892
Phone: (301) 402-0129
Email: lfox@niaid.nih.gov

NIAID Medical Monitor
Renee Browning, RN, MSN
Henry M. Jackson Foundation
DAIDS/NIAID/NIH
6700-B Rockledge Drive
Bethesda, MD 20892
Phone: (301) 435-3770
Email: browningr@niaid.nih.gov

NICHD Medical Officers
Lynne M. Mofenson, MD
Pediatric, Adolescent and Maternal AIDS Branch
National Institute of Child Health and Human Development (NICHD)
US National Institutes of Health
Rockville, MD 20852
Phone: (301) 435-6870
Email: LM65D@nih.gov
Email : Lynne.Mofenson@nih.hhs.gov

Heather Watts, MD
Pediatric, Adolescent and Maternal AIDS Branch
National Institute of Child Health and Human Development (NICHD)
US National Institutes of Health
Bethesda, MD 20892
Phone: (301) 435-6874
Email: wattsh@mail.nih.gov

Rohan Hazra, MD
NICHD/NIH
6100 Executive Boulevard
Bethesda, MD 20892-7510
Phone: (301) 435-6868
E-mail: hazrar@mail.nih.gov

Protocol Statisticians
Terrence Fenton, EdD
Principal Research Scientist
Statistical & Data Analysis Center
Harvard School of Public Health
651 Huntington Avenue
Boston, MA 02115-6017
Phone: (617) 632-2009
Email: fenton@sdac.harvard.edu

Li Liu, PhD
Center for Biostatistics in AIDS Research
Harvard School of Public Health
651 Huntington Avenue
Boston, MA 02115-6017
Phone: (617)432-1460
Email: lliu@sdac.harvard.edu

Laboratory Technologists
William B. Kabat, BS
Lab Manager
The Children's Memorial Hospital
Special Infectious Diseases Laboratory
2300 Children's Plaza, Box 82
Chicago, IL 60614-3394
Phone: (312) 227-6290
Email: bkabat@childrensmemorial.org

Amy Loftis, BS
Research Specialist
Retrovirology Core Lab
University of North Carolina at Chapel Hill1
16 Manning Drive
Chapel Hill, NC 27599
Phone: (919) 966-6867
Email: amy_james@med.unc.edu

Protocol Data Managers
Michael Basar, BS
Lead Data Manager
Frontier Science & Technology Research Foundation
4033 Maple Road
Amherst NY 14226-1056
Phone: (716) 834-0900 x7271
Email: basar.michael@fstrf.org

John Gaeddert, MPH
Data Manager
Frontier Science & Technology Research Foundation
Amherst, NY 14226-1056
Phone: (716) 834-0900 Ext. 7477
Email: gaeddert.john@fstrf.org

Linda Marillo, B.A.
Frontier Science & Technology Research Foundation
Amherst, NY 14226-1056
Phone: 716-834-0900
E-mail: marillo@fstrf.org

Linda Millar, BS
Co-Lead Data Manager
Frontier Science & Technology Research Foundation
4033 Maple Road
Amherst, NY 14226-1056
Phone: (716) 834-0900, ext. 7240
E-mail: millar.linda@fstrf.org

Protocol Pharmacist
Lynette Purdue, PharmD
National Institutes of Health
PAB/DAIDS/NIAID/NIH
6700-B Rockledge Drive MSC 7620
Bethesda, MD 20892-7620
Phone: (301) 435-3744
Email: lpurdue@niaid.nih.gov

Protocol Endocrinologist
Linda A DiMeglio, MD, MPH
Section of Pediatric Endocrinology and Diabetology
705 Riley Hospital Drive
Indiana University, School of Medicine
Indianapolis, IN 46202-5225
Phone: (317) 944-3889
E-mail: dimeglio@iupui.edu

Laboratory Data Coordinators
Amy Jennings, BS
Frontier Science & Technology Research Foundation
4033 Maple Road
Amherst, NY 14226
Phone: (716) 834-0900, Ext. 7438
Email: jennings@fstrf.org

Adam Manzella, MA
Frontier Science &Technology Research Foundation
4033 Maple Road
Amherst, NY 14226
Phone: (716) 834-0900, Ext. 7418
Email: manzella@fstrf.org

Amanda Zadzilka, BS
Frontier Science & Technology Research Foundation
4033 Maple Road
Amherst, NY 14226
Phone: (716) 834-0900, Ext. 7282
Email: zadzilka@fstrf.org

DXA Specialists

Heidi Kalkwarf, PhD, RD
Associate Professor of Pediatrics
University of Cincinnati College of Medicine
Cincinnati Children's Hospital Medical Center
3333 Burnet Avenue
Cincinnati, OH 45229
Phone: (513) 636-3803
Email: Heidi.kalkwarf@cchmc.org

John Shepherd, PhD
Assist Professor in Residence
Dept of Radiology and Bioimaging, Suite 525
University of California San Francisco
1635 Divisadero Street
San Francisco, CA 94115
Phone: (415) 885-7227
Email: john.shepherd@radiology.ucsf.edu

Pharmacologist

Mark Mirochnick, MD
Boston Medical Center
One Boston Medical Center Place
Boston, MA 02118
Phone: (617) 414-3754
Email: markm@bu.edu

Field Representatives

Michele A. Kelly, CPNP, NPP
(PROMISE) Stony Brook University Medical Center
Dept of Pediatric Infectious Diseases
HSC Level 11
Stony Brook, New York 11794-8111
Phone: (631) 444-8184
E-mail: mkelly@notes.cc.sunysb.edu

Mary Patricia Toye, RN, MS
Program Manager/Research Study Coordinator
Baystate Medical Center and Children's Hospital
759 Chestnut Street
Springfield, MA 01199
Phone: (413) 794-5399
E-mail: maripat.toye@bhs.org

Pharmaceutical Company Representatives

James F. Rooney, MD
Vice President, Medical Affairs
Gilead Sciences
333 Lakeside Drive
Foster City, CA 94404
Phone: (650) 522-5708
E-mail: jim.rooney@gilead.com

Oxana Ivanova
Gilead Sciences, Inc.
333 Lakeside Drive
Foster City, CA 94404
Phone: (650) 372-7656
Email: Oxana.Ivanova@gilead.com

Investigator

Patricia M. Flynn, MD
St. Jude Children's Research Hospital
Department of Infectious Disease
332 North Lauderdale Street
Memphis, TN 38105
Phone: (901) 495-2338
E-mail: pat.flynn@stjude.org

Allison L. Agwu, MD
Johns Hopkins University School of Medicine
Department of Pediatrics Division of Pediatric Infectious Diseases
200 North Wolfe Street
Baltimore, MD 21287
Phone: (410) 614-3917
Email: ageorg10@jhmi.edu

Protocol Specialist

Kathleen George, MPH
Family Health International
P.O. Box 13920
Durham, NC 27709
Phone: (919) 544-7040 X11150
E-mail: kgeorge@fhi360.org

TABLE OF CONTENTS

STUDY MANAGEMENT i
GLOSSARY ii
SCHEMA 1
1.0 INTRODUCTION 4
1.1 Background 4
1.2 Safety and Toxicity of Tenofovir 4
1.3 Rationale 14
2.0 STUDY OBJECTIVES 15
2.1 Primary Objectives 15
2.2 Secondary Objectives 16
2.3 Exploratory Objectives 17
3.0 STUDY DESIGN 17
4.0 SELECTION AND ENROLLMENT OF SUBJECTS 18
4.1 Antepartum Part of Substudy (TDF Exposure during Pregnancy) 19
4.2 Postpartum Part of Substudy (TDF Exposure during Breastfeeding) 19
4.3 Enrollment Procedures 20
5.0 STUDY TREATMENT 20
6.0 SUBJECT MANAGEMENT 21
6.1 Toxicity Management 21
6.2 Subject Management 21
6.3 Concomitant Medication Guidelines 21
6.4 Timing of Availability of Substudy Evaluation Results 21
6.5 Criteria for Treatment Discontinuation 22
6.6 Criteria for Study Discontinuation 22
7.0 EXPEDITED REPORTING OF SERIOUS ADVERSE EVENTS 22
8.0 STATISTICAL CONSIDERATIONS 22
8.1 General Design Issues 22
8.2 Outcome Measures 23
8.3 Randomization and Stratification 25
8.4 Sample Size and Accrual 25
8.5 Monitoring 29
8.6 Analyses 29
9.0 CLINCAL PHARMACOLOGY PLAN 30
9.1 Rationale 30
9.2 Pharmacology Objectives 30
9.3 Study Design, Modeling and Data Analysis 31
10.0 HUMAN SUBJECTS 32
10.1 Institutional Review Board and Informed Consent 32
10.2 Subject Confidentiality 33
10.3 Study Discontinuation 33
11.0 PUBLICATION OF RESEARCH FINDINGS 33
12.0 BIOHAZARD CONTAINMENT 33
13.0 REFERENCES 35
APPENDIX I: MATERNAL P1084S SCHEDULE OF EVALUATIONS 40
APPENDIX II: INFANT P1084S SCHEDULE OF EVALUATIONS 42
APPENDIX III: INFORMATION REGARDING RADIATION EXPOSURE FOR IRBS 44
APPENDIX IV: SAMPLE INFORMED CONSENT 45

STUDY MANAGEMENT

Email the Computer Support Group at the Data Management Center (DMC) (user.support@fstrf.org) to have relevant site personnel added to the protocol email group (promise.protP1084s@fstrf.org) immediately after completing protocol registration. Inclusion in the protocol e-mail group will ensure that sites receive important information about the study during its implementation and conduct.

General Questions: Email questions concerning any aspect of protocol interpretation and/or study implementation not listed below, including administrative, ethical, regulatory, clinical, counseling, data and laboratory operations, to promise.questions@fstrf.org.

Randomization/Registration: For randomization/registration questions or problems and study identification number (SID) lists, email rando.support@fstrf.org or call the DMC Randomization Desk at (716) 834-0900 x7301.

Computer and Screen Problems: For computer and screen problems, email user.support@fstrf.org or call the DMC at (716) 834-0900 x7302.

GLOSSARY

1077BF	Breastfeeding version of PROMISE
1077BA	Breastfeeding antepartum component of PROMISE
1077BP	Breastfeeding postpartum component of PROMISE
1077FF	Formula feeding version of PROMISE
1077FA	Formula feeding antepartum component of PROMISE
AGA	Appropriate for gestational age
AP	Antepartum
ARV	Antiretroviral therapy
BMC	Bone mineral content
BMD	Bone mineral density
BS ALP	bone-specific alkaline phosphatase
CBV	Combivir
CrCl	Creatinine clearance
D-pyr	Bone resorption (deoxypyridinoline)
DXA	Dual energy X-ray absorptiometry
FDA	U.S. Food and Drug Administration
GPAQ	Global Physical Activity Questionnaire
HAART	Highly active antiretroviral therapy
HBV	Hepatitis B virus
HepBSAg	Hepatitis B Surface Antigen
HIV	Human immunodeficiency virus
IMPAACT	International Maternal Pediatric Adolescent AIDS Clinical Trials Group
IGF	Insulin-like Growth Factor
LGA	Large for gestational age
LS	Lumbar Spine
LS BMD	Lumbar spine bone mineral density
LS BMC	Lumbar spine bone mineral content
MOP	Manual of Procedures
MREM	Millirem
NVP	Nevirapine
NTx	N-telopeptide
OHRP	Office for Human Research Protections
PP	Postpartum
PROMISE	Promoting Maternal and Infant Survival Everywhere Study
PTH	Parathyroid Hormone
RSC	DAIDS Regulatory Support Center
Scr	Serum Creatinine
SD	Single dose
SGA	Small for gestational age
SIP	Site Implementation Plan
s-CTx	serum C-terminal telopeptide of type 1 collagen
sdNVP	Single dose Nevirapine
TDF	Tenofovir disproxil fumarate (oral drug)
TFV	Tenofovir
TRV	Truvada
U.S.	United States
WB	Whole Body
WB BMC	Whole body bone mineral content
ZDV	Zidovudine

SCHEMA

IMPAACT P1084s
Maternal and Infant Monitoring for Evidence of Toxicity Related to Tenofovir Exposure: The Bone and Kidney Health Substudy of the 1077 PROMISE Protocol (Promoting Maternal and Infant Survival Everywhere)

DESIGN: Nested, comparative substudy of bone, renal and growth outcomes of subjects randomized to maternal tenofovir (TDF) or no maternal TDF during pregnancy or during breastfeeding in 1077BF and 1077FF

POPULATION: For AP exposure part of P1084s: Mother-infant pairs enrolled in the AP components of 1077BF or 1077FF (1077BA or 1077 FA, respectively) at African clinical sites approved as P1084s DXA sites.

For PP exposure part of P1084s: Mothers and their infants enrolled in the PP component of 1077BF (1077BP) at African clinical sites approved as P1084s DXA sites.

Note: Mother-infant pairs enrolled under Version 1.0 of the protocol will continue in follow-up through 74 weeks postpartum and undergo all visit procedures as indicated in the current protocol version. These mother-infant pairs will contribute to the primary and secondary analyses.

SAMPLE SIZE: 875 mother-infant pairs with 475 pairs enrolled in the AP part and 400 mother-infant pairs enrolled in the PP part.

STUDY DURATION: Mothers and infants will be followed for 74 weeks post-delivery.

PRIMARY OBJECTIVES:

Maternal:

AP exposure

1. To compare the renal and bone toxicity between the groups randomized to a TDF-containing triple ARV regimen vs. a Combivir (CBV)-containing triple ARV regimen during pregnancy, assessed as creatinine clearance (CrCl), bone resorption (Dpyr) and total lumbar spine bone mineral density (LS BMD) at delivery.

PP exposure

2. To compare the renal and bone toxicity between groups randomized to a maternal TDF-containing triple ARV regimen vs. infant NVP (no maternal ARVs) during breastfeeding, assessed as changes in CrCl and LS BMD from delivery to 74 weeks postpartum and Dpyr at 74 weeks.

Infant:

AP exposure

3. To compare the renal and bone toxicity between the infants, whose mothers are randomized to a TDF-containing triple ARV regimen vs. a CBV-containing triple ARV regimen during pregnancy, assessed as CrCl, Dpyr and bone mineral content (BMC) of both whole body (WB) and lumbar spine (LS) at delivery.

PP exposure

4. To compare the renal and bone toxicity between infants randomized to a maternal TDF-containing triple ARV regimen vs. infant NVP (no maternal ARVs) during breastfeeding, assessed as CrCl, Dpyr and LS BMC at 26 weeks of life.

AP and PP exposures

5. To compare fetal/infant somatic growth by TDF exposure status during pregnancy measured at delivery and during breastfeeding measured at 26 weeks of life, assessed as length-for-age Z-score, weight-for-age Z-score, and head-circumference Z-score, respectively.

SECONDARY OBJECTIVES:

Maternal:

AP exposure

1. To compare the renal and bone toxicity between the groups randomized to a TDF-containing triple ARV regimen vs. a CBV-containing triple regimen during pregnancy, assessed as: changes in CrCl and Dpyr from delivery to 6, 26, and 74 weeks postpartum; and hip BMD at delivery and changes in hip and LS BMD from delivery to 74 weeks postpartum.
2. To compare the renal and bone toxicity between the groups randomized to a TDF-containing triple ARV regimen vs. short-course ZDV with single dose nevirapine (sdNVP) during pregnancy, assessed as: CrCl, Dpyr and hip and LS BMD at delivery; changes in CrCl and Dpyr from delivery to 6, 26, and 74 weeks postpartum; and changes in hip and LS BMD from delivery to 74 weeks postpartum.

PP exposure

3. To compare the renal and bone toxicity between the groups randomized to a maternal TDF-containing triple ARV regimen vs. infant NVP (no-triple ARV regimen), assessed as: changes in CrCl and Dpyr from delivery to 6 and 26 weeks postpartum, and changes in hip BMD from delivery to 74 weeks postpartum.
4. To compare the mineral composition of breast milk from mothers taking TDF to that of breast milk from mothers not taking TDF.
5. To evaluate the relationship between women's hip and LS BMD change from baseline to 74 weeks postpartum and the duration of breastfeeding, and if that relationship differs by TDF exposure category.

AP and PP exposure

6. To compare the renal and bone toxicity by TDF exposure during pregnancy and breastfeeding, assessed as changes in CrCl and Dpyr (if available) at delivery and 6, 26, and 74 weeks postpartum and changes in hip and LS BMD from delivery to 74 weeks postpartum

PK analyses

7. To estimate maternal TFV pharmacokinetic parameters and drug exposure among women who take TDF during pregnancy, delivery and postpartum and to correlate maternal TFV exposure with markers for renal and bone toxicity.
8. To characterize the kinetics of TFV transfer from maternal plasma to breast milk and estimate infant breast milk TFV dose.

Infant:

AP exposure

9. To compare the renal and bone toxicity between the groups whose mothers were randomized to a TDF-containing triple ARV regimen vs. a CBV-containing triple ARV regimen during pregnancy, assessed as changes in CrCl and Dpyr from delivery to 10, 26 and 74 weeks of life and in LS BMC from delivery to 26 weeks of life.
10. To describe the concentrations of hormonal growth factors in infants in relation to infant growth percentile and antepartum TDF exposure status at birth, 10 and 74 weeks of life.
11. To compare the renal and bone toxicity between the infants whose mothers are randomized to a TDF-containing triple ARV regimen vs. short-course ZDV with sdNVP during pregnancy, assessed as: CrCl, Dpyr, and BMC of both WB and LS at delivery; changes in CrCl and Dpyr from delivery to 10, 26 and 74 weeks of life; and changes in LS BMC from delivery to 26 weeks of life.

PP exposure

12. To compare the renal and bone toxicity between the groups randomized to a maternal TDF-containing triple ARV regimen vs. infant NVP (no-triple ARV regimen), assessed as changes in CrCl and Dpyr from delivery to 10 and 74 weeks of life.
13. To estimate infant TFV exposure from breast milk using the estimated infant breast milk drug dose, measured infant plasma drug concentrations and population modeling.

AP and PP exposure

14. To compare the renal and bone toxicity by maternal TDF exposure during pregnancy and breastfeeding assessed as CrCl and Dpyr (if available) at delivery and weeks 10, 26, and 74 of life and LS BMC at delivery and week 26 of life.

1.0 INTRODUCTION

1.1 Background

General Description and Pharmacokinetics of Tenofovir:
Tenofovir is an acyclic nucleotide analogue with activity against retroviruses, including HIV-1, HIV-2 and hepatitis B virus (1). The highly charged anionic nature of TFV, however, results in low bioavailability. *TFV will be used throughout this document to represent this anionic, relatively non-bioavailable form of the drug.* Tenofovir disoproxil fumarate is an orally active ester prodrug of tenofovir, approved for treatment of HIV-infection in adults; *TDF will be used throughout this document to represent this approved, oral form of the drug.* TDF is rapidly hydrolyzed to TFV by plasma esterases, then metabolized intracellularly to the active drug (tenofovir diphosphate), which competitively inhibits the HIV RT enzyme and terminates the DNA synthesis. Oral bioavailability in adults ranges from 25% (fasting) to 39% (after a high-fat meal). TFV is excreted unchanged by the kidneys by a combination of glomerular filtration and active tubular secretion; TFV plasma clearance and exposure are related to the body weight/serum creatinine ratio (BW/SCR) (2). TDF is pregnancy category B and is not classified as a teratogen.

1.2 Safety and Toxicity of Tenofovir

The safety data on TDF (alone or in coformulation with emtricitabine (FTC)) is primarily from adult treatment trial data and broad clinical experience in the US. The main toxicities include bone demineralization and renal toxicity (especially tubular dysfunction). The adult safety data are fully summarized in the antepartum section (Section 2.0) of the 1077BF version of the PROMISE protocol.

1.2.1 Renal Toxicity

Animal Studies:
Organic anion transporters in the proximal tubules take up TFV (and related compounds cidofovir and adefovir) and the accumulation in the tubular cells causes dose-limiting toxicity in animal studies (3). As a result, renal insufficiency and proximal tubular dysfunction are the expected manifestations of TFV nephrotoxicity (3). Signs of this reversible renal toxicity can include increases in serum creatinine, blood urea nitrogen (BUN), glycosuria, proteinuria, phosphaturia, and calciuria and decreases in serum phosphate (Fanconi syndrome), as observed in animal studies at high exposure levels (30 mg/kg/day) (4). In severe cases, proximal renal tubular dysfunction leads to hyperphosphaturia with hypophosphatemia and increased bone resorption, producing abnormal bone mineral loss analogous to that seen in hypophosphatemic rickets; these changes were at least partially reversible with dose reduction or discontinuation (4). Renal toxicity was not observed in infant macaques treated with low-dose TDF (10 mg/kg/day, approximately 3 times the human exposure following a dose of 300 mg TDF) for 5 years (4).

Adult Human Data:
Clinically significant TDF-associated renal toxicity has been observed infrequently in prospective and retrospective clinical studies of adults (5-11). Review of changes in renal parameters in over 1,000 adults in randomized trials revealed statistically significant but very small decrements in glomerular filtration rate (GFR) in TDF patients compared

to non-TDF patients over 3 years; there was no evidence of clinically significant renal toxicity (12). Cases of nephrotoxicity (Fanconi syndrome including hypophosphatemia, renal insufficiency, acute tubular necrosis, acute renal failure) have been reported in adults receiving TDF in combination with other drugs (13-18). Tubular dysfunction in the absence of decline in GFR appears to occur more frequently than frank renal insufficiency (19). Chart review of 16 pregnancies in which TDF was prescribed revealed one woman with transient decrease in GFR below 90 mL/minute/1.72m^2 that resolved without discontinuation of TDF therapy (20).

Pediatric Data:
Renal insufficiency and Fanconi syndrome associated with TDF use have been reported in perinatally infected children (21, 22). TDF has been associated with renal dysfunction in US and UK-based cohort studies (23, 24). There is scant experience with administration of TDF in infants, making it difficult to estimate the relative likelihood of TDF-related renal toxicity in infants. There was no reported renal toxicity in infants in a study in which single dose TDF for PMTCT was given to mothers in labor and then to their newborns (25). Similarly, there has been no evidence of infant renal toxicity in study HPTN 057, in which mothers received TDF in labor and/or newborns received up to 3 doses of TDF in the first week of life (26). Chart review of 15 infants born to women taking TDF during pregnancy did not reveal evidence of clinically detected renal or growth problems (20). Newborns may be less susceptible to renal toxicity from TDF because organic anion transporter (OAT) mechanisms (responsible for uptake of TFV from plasma) are poorly developed at birth (3). However, in the sheep model, OAT function is relatively mature by the time of birth (27).

Assessment of Renal Function and Toxicity:
Impaired renal function may be reflected in a reduction in the glomerular filtration rate (GFR), which is commonly estimated from creatinine clearance. Serum creatinine is often used to estimate GFR, but dramatic reductions in renal function may have already taken place by the time creatinine rises, making it a relatively insensitive marker, especially for mild to moderate reductions in renal function. In children, a reasonable estimate of GFR can be calculated from the serum creatinine (Scr), height (L) and age-dependent proportionality constant (k) using the Schwartz formula: Estimated GFR (mL/min/1.73 m^2) = kL/Scr (28).

In adults, the creatinine clearance is most commonly measured using the Cockroft-Gault equation, adjusted for gender:

MEN: (140-age) x (weight in kg)/(72 x Scr)
WOMEN: (140-age) x (weight in kg) x 0.85/(72 x Scr) (29)

A recent study of renal function in African adults initiating HAART suggested that the Cockroft-Gault equation was valid in that population and appeared to perform better than an alternative GFR estimating equation for adults, the Modification of Diet in Renal Disease (MDRD) formula (30).

In pregnancy, GFR normally rises dramatically in the third trimester, and so different norms for GFR are expected.

Proximal tubular dysfunction: Proximal tubular dysfunction results in impaired reabsorption (and therefore increased urinary levels) of electrolytes, phosphate, glucose

and amino acids, and, when severe enough, can produce hypophosphatemia. Urine can be tested for presence of glucose (in the absence of hyperglycemia) or protein. In addition, the concomitant measurements of plasma and urinary phosphate, calcium, electrolytes, and amino acids can be used to assess the percentage of tubular reabsorption (TR) as a measure of proximal tubular dysfunction. Based on human and animal studies and postulated mechanisms related to bone toxicity, it will be important to calculate this parameter for phosphate and calcium. The formula for TR of phosphate (based on a spot urine collection), for example, is:

TR-phosphate% = {1 – [(U-phos/P-phos)/(Ucreat/P-creat)]} x 100%
[HLHB 17, p. 495]

1.2.2 Bone Toxicity

Animal Data:
Tenofovir and TDF administered orally in toxicology studies to rats, dogs, and monkeys at exposures (based on AUCs) $\geq$ 6-fold those observed in humans caused bone toxicity.[TDF package insert] In monkeys bone toxicity was diagnosed as osteomalacia. Osteomalacia observed in some monkeys appeared to be reversible upon dose reduction or discontinuation of tenofovir. In rats and dogs, bone toxicity manifested as reduced bone mineral density.

In animal studies of tenofovir, bone toxicity (like renal toxicity) was more likely with higher TFV exposures and longer duration of treatment. These bone changes occurred in animals at high doses (subcutaneous TFV 30 mg/kg/day) and were associated with growth retardation, osteomalacia, increased risk of fractures (especially in growing bones), increased serum alkaline phosphatase, and decreased serum phosphorus (4, 31). Taken together, these findings suggest that bone demineralization may result from TDF-related perturbations of phosphate metabolism, including inhibition of intestinal phosphate absorption and renal tubular phosphate reabsorption (4). Low bone mineral density (BMD) and bone mineral content (BMC) were not seen in TFV-treated infant macaques without proximal tubular dysfunction; however, bone mineral deficiencies were seen in 50% of these infant macaques with proximal renal tubular dysfunction, and BMD and BMC improved but did not normalize even after TFV dose reduction and mineral supplementation (3). Infant macaques receiving low daily doses of TFV (which were still higher than human exposures following 300 mg TDF) for 5 years, on the other hand, experienced normal growth and bone density (4).

Adult Human Data:
The issue of potential TDF bone toxicity in human studies is complicated by the finding that HIV-infected patients are already at increased risk of low BMD and other bone problems.

Available data from adults suggest that multiple factors contribute to BMD loss in HIV-infected men and women, that greater immunosuppression at the time of ARV initiation may be a risk factor for greater BMD loss, and that while initiation of any ARV regimen may be associated with loss of BMD at least for the first 1-2 years, the effect of TDF on BMD may be greater. Fracture incidence appears to be higher in HIV-infected adults but there is conflicting evidence about the specific role of TDF in increasing fracture risk (32-34).

Pediatric Data:
Bone toxicity in children must be interpreted in the developmental context that, throughout childhood, bones must normally grow (and therefore turnover), mature and adequately mineralize in order to reach adult size and quality. Thus, bone density, turnover, strength and other factors cannot be compared against a single normal standard but rather against norms that are adjusted for age and other demographic factors.

Like HIV-infected adults, HIV-infected children and youth have lower BMD than would be expected for healthy people of similar age, weight and race (35-38).

While complete understanding of the many factors contributing to abnormal BMD in HIV infection is lacking, major risk factors that have emerged from longitudinal studies include longer duration of HIV disease, low BMI, history of weight loss, and previous use of steroids. TDF therapy has also emerged as a specific concern for loss of BMD in children.

In general, longitudinal studies have shown that initiation of HAART is associated with an improvement from low baseline BMD over time. Mora et al., in a study of 32 perinatally infected children receiving PI-based, non-TDF-containing HAART, demonstrated low BMD with biochemical evidence of increased bone turnover (increased formation and resorption) at baseline; BMD improved but bone turnover did not normalize during the one year duration of the study (36).

Jacobsen et al. studied 37 HIV-infected children compared to nine HIV uninfected sibling controls (39). When compared with population norms, HIV-infected children had lower than expected age- and sex-adjusted bone mass. Independent predictors of low BMD in HIV-infected children suggested contributions from delays in growth and sexual maturity, duration of HIV infection, ethnicity and disease severity. Multivitamin use was strongly associated with better bone mineral density, but not with changes in BMD over time. Change in BMD Z-score over time showed that BMD was stable or increased in controls but stable/increased BMD was observed in only 44% of the HIV-infected children, a difference that approached statistical significance (39).

While the association in cross-sectional studies between PIs and low BMD has not been consistently confirmed in longitudinal studies, the potential effect of TDF may be more concerning. As mentioned, initiation of tenofovir-containing HAART has been associated with a greater initial decrease in BMD than non-TDF-containing HAART in adults (40); initiation of tenofovir-containing HAART has likewise been associated with loss of BMD in children (41). In adults, the initial loss seems to stabilize over time, but the pattern is less clear in children. The effect of tenofovir may be partly attributable to its ability to cause a Fanconi-like syndrome with renal phosphate wasting and secondarily increased osteomalacia and bone resorption. The effects may be exaggerated in younger children, who may be receiving a disproportionately high dose of TDF. Hazra et al. treated sixteen children and adolescents with perinatally-acquired HIV infection with TDF and at least three other ARTs as part of a salvage regimen (42). Five of 15 evaluated at 48 weeks had experienced a greater than 6% decline in their absolute LS BMD despite linear growth, resulting in worse Z-scores than when they started the trial (since BMD would be expected to increase over time at these ages).

While the Hazra/Gafni studies involved highly ARV experienced children, another study looking at 16 children with well-controlled HIV infection undergoing a switch from d4T

to tenofovir failed to show an effect of TDF on BMD (43). In a study of adolescents with virologic failure randomly assigned to a new regimen with TDF vs. TDF-placebo, there was a trend (p=0.1) for a greater proportion in the TDF group (18%) than in the placebo group (3%) to have $\geq$ 4% loss in spine BMD (44).

Thus, low BMD appears to be relatively common in HIV-infected children. In addition to the traditional risk factors for low BMD unrelated to HIV that may be present in these patients, antiretroviral treatment appears to confer an increased risk. It is not clear if the lower BMD observed in HIV-infected children confers a substantial increase in risk of fracture (45).

The bone toxicity observed in infant macaques given high doses of tenofovir raises concerns about greater susceptibility to this toxicity in human infants. There is scant experience, however, with administration of TDF in infants, making it difficult to estimate the relative likelihood of TDF-related bone toxicity in infants. There has been no evidence of infant bone toxicity – as monitored by plain films in the first week of life and at 6 months of life – in study HPTN 057, in which mothers received TDF in labor and/or newborns received up to three doses of TDF in the first week of life (46).

1.2.3 Pregnancy/Fetal Exposure

Animal Data:
Studies of daily subcutaneous administration of TFV (at high doses of 30 mg/kg subcutaneously) to pregnant Rhesus macaques reported a fetal/maternal concentration of 8.5%, with maternal levels peaking at 30 minutes and fetal levels peaking at 1-3 hours after dosing. Even though maternal levels were lower later in pregnancy, fetal levels were proportionately higher. Newborns exposed to TFV transplacentally had low birth weights; ongoing growth restriction, hypophosphatemia, increased bone-specific alkaline phosphatase and development of skeletal abnormalities were common (about 25%) but all animals received postnatal TFV in addition to antepartum exposure (47).

Chronic exposure of fetal monkeys to TFV at a high dose of 30 mg/kg subcutaneously (25 times the AUC levels achieved with therapeutic doses in humans) from days 20-150 of gestation did not result in gross structural abnormalities on fetal necropsy (47). However, significantly lower fetal circulating insulin-like growth factor 1 (IGF-1) levels were reported and were associated with fetal weights 13% lower than untreated controls. A slight reduction in fetal bone porosity was also observed within 2 months of maternal treatment (46). Because the fetus depends upon the mother as its source of phosphate and other minerals, the effects on the fetus of maternal administration of TDF may be mediated through maternal renal toxicity and hypophosphatemia (4). In fact, TFV-treated pregnant rhesus monkeys had biomarker evidence of increased bone turnover (47).

Long-term, lower doses of TFV did not affect the long-term outcomes of offspring of a macaque treated lifelong, including throughout pregnancies, with TFV (3).

Clinical Data:
In pregnant women in labor, a single dose of 300 mg of TDF resulted in maternal TFV concentrations comparable to those seen in non-pregnant adults after a single 300 mg dose (48). In two studies of pregnant women, the cord-to-maternal blood ratio ranged from .95 to .99 indicating high placental transfer though 60% placental transfer has also

been reported (48). In a more recent study, single 300 mg TDF dose to women in labor resulted in average TFV concentration of 69 ng/mL in mothers at delivery, of 31ng/mL in cord blood, and an overall ratio of cord blood to maternal delivery blood of 0.44 (49).

TFV pharmacokinetics with chronic administration during pregnancy has been studied in 19 HIV-infected pregnant women. TFV AUC, peak and trough concentrations were lower during the third trimester of pregnancy compared to postpartum, but these differences do not appear to be of sufficient magnitude to require dosing modification during pregnancy. The median ratio of cord blood to maternal delivery blood was 1.04 with a range of 0.6 to 1.7. Of 16 women who had HIV viral load measured during the third trimester, 14 were undetectable (< 400 copies/mL) (50).

In a recent case series, only 2 of 76 pregnant women taking TDF stopped TDF for toxicity (one for rash and one for nausea). All 78 infants were healthy with no signs of toxicity, and all were HIV uninfected. However, maternal creatinine was the only measure reported for monitoring renal toxicity and no specific bone toxicity monitoring for mothers or infants was reported. In addition, the change in the study population average and range of creatinine from baseline [0.58 (0.3-0.9)] to time of delivery [0.63 (0.4-1.1)] could represent the subtle increase in creatinine seen with evolving TDF toxicity.

Chart review of 16 pregnancies in which TDF was prescribed revealed one woman with transient decrease in GFR below 90 mL/minute/1.72 m^2 that resolved without discontinuation of TDF therapy. Complications in nine pregnancies (including one spontaneous abortion) were not attributed to TDF exposure.(20) Chart review of 15 live-born infants born to women in this study did not reveal evidence of clinically apparent renal, growth or bone problems (20).

Despite marked increase in use of TDF by HIV-infected pregnant women (51), evidence of clinically important adverse fetal/infants effects have not been reported. Analysis of early infant growth outcomes after maternal TDF use in a large US cohort found no evidence of adverse fetal growth effects after maternal TDF use (52). That same study reported slightly smaller lengths, head circumferences in one-year old infants whose mothers received TDF during pregnancy, but the clinical significance of these differences is not clear, and there were no differences in major growth outcomes at age one year by maternal TDF use. In a separate study, TDF use during pregnancy was not associated with growth impairment or bone density reductions in a smaller cohort of HIV-exposed children evaluated by quantitative ultrasound) (53).

1.2.4 Potential Risk to Mother and Infant during Lactation and Breastfeeding

After administration of single-dose TFV 30 mg/kg to two lactating Rhesus macaques, breast milk peak and AUC levels were 2-4% and 20%, respectively, of maternal levels (54). Oral administration of TDF and other TFV prodrugs to beagles results in rapid conversion in serum to TFV, the form in which the drug would be expected to be excreted in breast milk (54). The highly charged anionic nature of TFV results in low bioavailability: 6% in rats, 18% in dogs and 5% in cynomolgus monkeys. There is limited information describing TFV concentration in human breast milk. TFV was detectable in low concentrations (6.3 – 17.8 ng/mL) in breast milk collected on day 1 or 2 of life from 3 of 4 women who received single 600 mg doses prior to delivery (26). A study that included women who received 300mg daily TDF for one week following

600mg TDF in labor found similar breast milk concentrations (55). Neither study measured the TFV concentration in the exposed infants, but the presence of the less bioavailable TFV (as opposed to TDF) at these levels predicts low effective exposure levels to TFV for breastfeeding infants.

Clinical Data and Concerns:
There is no published information about the potential for TDF-induced hyperphosphaturia with hypophosphatemia to alter the mineral composition of breast milk, which could lead to indirect negative effects on nursing infant bone health and growth. Inadequate phosphate in preterm breast milk is a known contributor to poor bone mineralization (hypophosphatemic rickets) in preterm infants fed unfortified breast milk. High capacity active transport mechanisms allow for efficient transport of large amounts of calcium and phosphate into breast milk (56). Mechanisms of phosphate transport are incompletely understood and appear to be multiple, including 1) a sodium-dependent Na-P cotransport similar to that found in kidneys but less susceptible to inhibition by substrate analogues, and 2) a less well characterized anion-exchange transport mechanism (56). It is not known if the anionic TFV would be transported into milk by such a mechanism or whether TFV could interfere with phosphate transport by this mechanism. In addition, in lactating women, renal reabsorption of phosphate is normally *increased;* thus, TDF-related hyperphosphaturia (due to disturbance of the proximal tubular reabsorption of phosphate by TFV) may exaggerate urinary phosphate losses at the very time when a woman needs increased conservation of phosphate for lactation, and the resulting phosphate deficit may be compensated by increasing bone resorption (57). Furthermore, phosphate transport into milk also depends on concerted action by cortisol, insulin and prolactin; TDF, other ARVs, or other factors common in the study population that affect these hormones may also affect transfer of phosphate into breast milk (58). Phosphate secretion into milk rises dramatically beginning at about 36 hours postpartum; thus TFV levels in breast milk may be different before and after this 36 hour postpartum period (59).

1.2.5 Pregnancy, Lactation, Hepatitis B Infection and Other Factors Affecting Bone Health

To assess the potential bone and renal toxicity attributable to TDF in the PROMISE study, it will be necessary to collect data about other factors that can result in similar toxicities. In particular, general malnutrition, lower activity level, vitamin D deficiency, inadequate calcium or phosphorous intake, endocrinopathies, and concomitant HIV- and non HIV-medications (corticosteroids, anticonvulsants, hormonal contraceptives) in the mother or the infant, recent or ongoing pregnancy/lactation in the mother, and preterm birth of infant may contribute to bone toxicity (60).

In normal pregnancy, the increased calcium demands from the fetus are met by increased maternal intestinal calcium absorption (57). Changes during pregnancy in biochemical markers related to mineral homeostasis include: decreased serum calcium but unchanged ionized calcium, increased urinary excretion of calcium (from 160 mg/day to 240 mg/day), decreased PTH, increased 1,25$(OH)_2$D (mediating increased intestinal absorption from 25% pre-pregnancy to 50% during pregnancy), increased parathyroid hormone (PTH)-related protein (PTHrp), increased alkaline phosphatase, and increased markers of bone resorption. While there may be about a 3% decline in bone density in the spine and pelvic bone (trabecular bone), there is a simultaneous increase in cortical bone density that results in a net zero change in bone mineral content during pregnancy.

However, pregnant women with deficient calcium intake may lose 2-3% of bone mineral content with proportionately larger bone mineral losses in areas of trabecular bone, such as the lumbar spine and pelvis (61).

During lactation, however, the large amount of calcium required for breast milk is derived largely from maternal bone mineral resorption. Histomorphometric data from animals demonstrate increased bone turnover and losses of 30% of bone mineral during the animal's normal lactation period (57). In healthy women, bone density during lactation declines of 3% to 10% in bone mineral content after 2 to 6 months of lactation, especially at trabecular sites (lumbar spine, hip, femur and distal radius) (57). Other studies reported an average of 5% spine BMD loss at 6 months (61). Bone mineral loss can reach 1% to 3% *per month* in lactating women, much greater than the *annual* loss 1%-3% in women with postmenopausal osteoporosis (57). Over the course of 12 months of lactation, 5-10% loss of BMD at the lumbar spine would be expected (61). Since breast milk calcium is preserved at the expense of maternal skeleton, however, mothers with insufficient calcium intake would be at risk of larger BMD losses (61). Increased bone turnover, due predominantly to increased bone resorption and likely mediated by PTHrp secreted by the mammary gland, continues throughout lactation and is reflected in persistence of pregnancy levels of bone turnover markers through lactation; conversely, 1,25$(OH)_2$D levels and intestinal calcium absorption revert to prepregnancy levels (61). It seems reasonable to estimate spine BMD losses of at least 10% during 12 months of lactation in women living in low resource settings, suffering from chronic illness, and lacking optimal nutrition; perhaps losses as high as 20-30% may occur. Maternal skeletal recovery after 3-6 months of breastfeeding is generally achieved by about 6 months after cessation of breastfeeding, but there are no firm data about the time to skeletal recover after periods of 12 months or longer of breastfeeding (61). Thus, lactation is a period of heightened susceptibility to lower bone mineral density (BMD), especially for women who have other risk factors for low baseline BMD or ongoing loss of BMD.

Chronic hepatitis B infection (as well as other causes of chronic hepatitis) is associated with derangements in normal bone turnover. Low BMD and even osteoporosis occur at high rates among adults with cirrhotic liver disease secondary to hepatitis B infection. There is some suggestion that low BMD, decreased bone formation and increased bone resorption may be common even among adults with chronic hepatitis B that has not progressed to cirrhosis, but increased bone turnover and bone loss risk in most studies has been predominantly observed in those with more advanced liver involvement (e.g., fibrosis, cirrhosis) with hepatitis B infection (62-64). The treatment of chronic hepatitis B infection with immunomodulatory agents (e.g., interferons) may have additional effects but are not considered here since such treatment is not anticipated in the clinical sites involved in this study.

1.2.6 Assessment of Bone Turnover and Bone Density

<u>Growth Impairment</u>:
Serial assessment of linear growth will be important, as growth was impaired in some animal fetuses and infants exposed to long-term, often high doses of TFV. In addition, the IGF system is established as the main regulator of fetal growth; suppression of IGF-1 was reported in small-for-gestational-age infant monkeys exposed to TFV in utero, suggesting that this may play a role in the pathophysiology of TDF-related impairment of fetal growth (65).

Markers of Bone Turnover:
Bone turnover reflects the balance of bone formation and bone resorption. Previous investigators have demonstrated that high bone turnover is an independent risk factor for hip fractures in older women (65). Although it is unlikely that young women will experience fractures, they may experience bone loss, a major endpoint in the substudy. Greenspan and others have previously demonstrated that bone turnover (bone resorption coupled with bone formation) is associated with the rate of bone loss in women (67-69). Lactating women, in particular, have dramatic increases in bone turnover due to increases in bone resorption (57).

Markers of bone resorption include urine deoxypyridinoline (dPYR)/urine creatinine ratio, urine N-telopeptide/urine creatinine ratio and s-CTx (C-terminal telopeptide of type 1 collagen). Markers of bone formation include P1NP (N-propeptide of type 1 collagen), osteocalcin and BSAP (bone specific alkaline phosphatase). When there is a decrease in BMD, it is important to determine whether this is due to an increase in bone resorption (increase in dPYR, s-CTx), a decrease in bone formation (P1NP, osteocalcin and BSAP), or possibly an increase in all markers, characterized by greater resorption than formation, leading to the loss of bone.

The main limitation will be that bone turnover (formation and resorption) is generally increased in the setting of rapidly growing bones in normal infants and children. However, clinical care still relies on many of these measurements. This limitation may be overcome by use of outcome measure of difference in levels of these markers between TDF-exposed subjects and controls although the clinical relevance of observed differences may not be clear.

Increased proinflammatory cytokines, such as IL-1 and TNF-alpha, have also been implicated in explaining how bone density loss due to increased bone resorption occurs in conditions (including HIV infection) with increase in these inflammatory cytokines (70).

Dual Energy X-ray Absorptiometry (DXA) Assessment of Bone Mineral Density:
DXA is the standard modality for bone assessment of adults. The radiation exposure with DXA is low (< 5 mrem), less than the exposure of a plain radiograph of the chest.

In a clinical setting, DXA BMD results are typically compared to the average bone density of young, healthy adult peak bone mass (T-score) and to the average bone density of other people of the same age, sex, and race (Z-score). For women, the classification of osteoporosis using DXA measurements of BMD is currently based on the T-score if they are age 30 or older. For women under age 30, the Z-score is used (comparison to age-matched) since peak bone mass has not yet been reached. Usually, 1 SD equals 10 to 15 percent of the BMD value in g/cm^2. Women lose 3-5% of their bone mass annually for the first 5 years after menopause, the condition best studied for its relationship to bone mineral loss and for which standardized calculations of fracture risks are available, based on BMD results together with demographic and clinical data.(National Osteoporosis Foundation, 2002) In premenopausal women (and children), the fracture risk associated with low BMD measurements is not well established, but Z-score < -2.0 is categorized as abnormally low BMD. Many experts regard a change or difference in BMD of 3-5% (or 0.5SD) as significant in these younger patients in whom standardized outcome data are lacking [*Personal communications: Todd Brown, Johns Hopkins University; Catherine Gordon, Boston Children's Hospital; Heidi Kalkwarf, Cincinnati Children's Hospital Medical Center*].

FIGURE 3. Z- and T-Scores

Bone Mineral Density

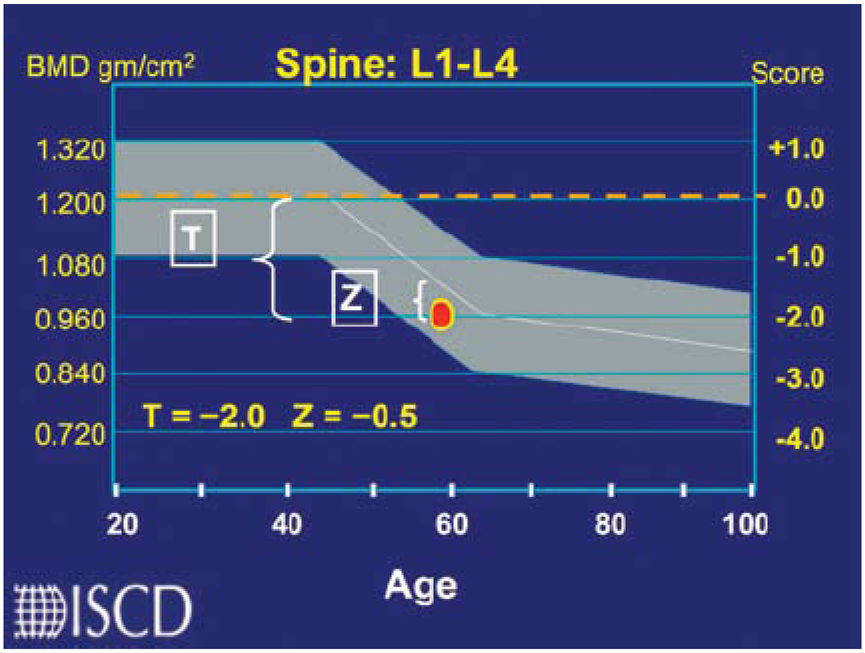

From: ISCD Bone Densitometry Clinician Course. Lecture 5 (2008).

Measurement and Classification:
The difference between the patient's score and the norm is expressed in standard deviations (SD) above or below the mean. Usually, 1 SD equals 10 to 15 percent of the BMD value in g/cm^2. Depending upon the skeletal site, a decline in BMD expressed in absolute terms (g/cm^2) or in standard deviations (T-scores or Z-scores) begins during young adulthood, accelerates in women at menopause and continues to progress in postmenopausal women and men age 50 and older (see Figure 3). (From http://www.nof.org/professionals/NOF_Clinicians_Guide.pdf)

Dual-energy x-ray absorptiometry (DXA) measurement of the hip and spine is the technology now used to establish or confirm a diagnosis of osteoporosis, predict future fracture risk and monitor patients by performing serial assessments. BMD is expressed in absolute terms of grams of mineral per square centimeter scanned (g/cm^2) and as a relationship to two norms:

- compared to the expected BMD for the patient's age and sex (Z-score), or
- compared to "young normal" adults of the same sex (T-score).

Normative data for DXA scan assessment of BMD in children have been developed for children as young as 6 years old in an NICHD-sponsored study; these standards are limited to late-model Hologic scanners (71). DXA is the preferred method for assessment of bone density in children with diseases that may affect bone health (72). However, there are NO standardized norms for interpretation of DXA in infants and young children.

DXA has been used to assess bone density and bone mineral content of infants, especially preterm infants, who are at risk of impaired bone mineralization (73). There are no standardized norms for infants, and there is concern that previous studies invalidating DXA for bone mineral assessment in infants used older "pencil-beam" DXA technology

that may not be directly applicable to newer “fan-beam” technology. Newer-technology DXA (Hologic QDR4500A) was used in preterm French infants (DXA performed at hospital discharge, and 2 and 4 months after discharge) to demonstrate significantly higher whole-body bone mineral content attainment in preterm very low birth weight infants fed an enriched preterm formula at discharge compared to similar infants fed a standard term formula (74). In a cross-sectional study in Turkey, a Hologic QDR 4500Eilte DXA scanner was used to assess BMD and BMC in SGA, AGA and LGA newborns within 24 hours of birth, and results showed that whole-body BMD and BMC measurements were significantly different by category of relative birth weight (LGA > AGA > SGA) (75). Furthermore, DXA was able to demonstrate bone mineral differences among the groups of infants, even though serum calcium, phosphorous and alkaline phosphatase measurements were not different among groups. Thus, while there may not be reference standards that establish normal and abnormal values for DXA in infants, DXA has been successfully used for comparing bone mineral status between different treatment groups or categories of infants (whole body BMC and BMD by late-model Hologic DXA).

The IMPAACT P1084s clinical research sites that have been approved to perform DXA scans for the study include Uganda, Zimbabwe, Malawi and South Africa. Additional sites may be added as needed.

1.3 Rationale

For many women, TDF may be an effective and well-tolerated part of a combination ARV regimen that treats maternal illness (HIV, HBV or both) and prevents maternal-to-child transmission antepartum, perinatally and through breast milk.

The effects of prolonged maternal TDF use on pregnant women and their infants have not been studied. Based on animal and non-pregnant human studies, the potential TDF toxicities of greatest concern are renal toxicity and bone toxicity and fetal/infant growth restriction. The maternal toxicity would be expected to be mediated by direct exposure to tenofovir in combination with other HIV-related and HIV-unrelated maternal factors related to renal toxicity and bone toxicity. The infant toxicity may be mediated through direct exposure to tenofovir through transplacental passage or breast milk transfer; however, infant toxicity may also be mediated by TDF-related alterations in maternal physiology (e.g., maternal hypophosphatemia) and potential TDF-related alterations in breast milk (e.g., alteration in phosphate concentration). In addition, other HIV-related and HIV-unrelated factors may contribute to problems with infant renal function and bone health. Finally, it is possible that neither antepartum nor postnatal TDF alone will lead to significant toxicity, but that use of TDF through pregnancy and postnatally will have additive or cumulative toxicity of greater significance.

The PROMISE study offers an opportunity to evaluate in more detail the safety of TDF-containing triple ARV regimens in pregnancy compared to non –TDF -containing ARV regimens (e.g., ZDV-containing regimens and non-triple ARV regimens) that are currently more commonly used. Since HIV+ pregnant women will be randomly assigned to TDF -containing and non-TDF-containing ARV regimens, antepartum enrollment of these women in this study will allow for further evaluation of their renal function, bone turnover and bone density and thus assess the potential differences due to TDF. In addition, the infants of these women can be assessed for differential effects of antepartum TDF vs. no TDF on infant growth, on baseline bone status, and on baseline renal status. (The comparison of rates of anemia between TDF and non-TDF groups

in this substudy will be an objective covered in the main PROMISE study and therefore not repeated here.)

Since eligible breastfeeding women in the postpartum component of the PROMISE study will be randomized to TDF-containing triple ARV regimen (either to continue the triple ARV regimen initiated during pregnancy or to initiate a triple ARV regimen) or no maternal ARV, enrollment of these women in this study will allow for evaluation of renal function, bone turnover and bone density in the women and their infants and infant growth during and after the breastfeeding period. Given that lactation is known to produce a substantial loss in maternal BMD, it will be particularly important to assess effects on BMD in women during this period of higher vulnerability to bone loss and to compare the amount of BMD loss in TDF vs. non-TDF groups of women in order to determine what proportion may be due to TDF vs. due to lactation and other factors.

The assessment of TFV levels in breastfeeding women, breast milk and infants as part of the main study will be important to quantify the direct exposure of these infants to TFV. In addition, assessment of breast milk mineral composition and infant growth and bone health over time in both the TDF and non-TDF maternal-infant pairs will permit the assessment of indirect effects of maternal TDF on infants due to altered renal function and mineral homeostasis due to TDF in the lactating mother.

2.0 STUDY OBJECTIVES

2.1 Primary Objectives

Maternal:

AP exposure

1. To compare the renal and bone toxicity between the groups randomized to a TDF-containing triple ARV regimen vs. a Combivir (CBV)-containing triple ARV regimen during pregnancy, assessed as creatinine clearance (CrCl), bone resorption (Dpyr) and total lumbar spine bone mineral density (LS BMD) at delivery.

PP exposure

2. To compare the renal and bone toxicity between groups randomized to a maternal TDF-containing triple ARV regimen vs. infant NVP (no maternal ARVs) during breastfeeding, assessed as changes in CrCl and LS BMD from delivery to 74 weeks postpartum and Dpyr at 74 weeks.

Infant:

AP exposure

3. To compare the renal and bone toxicity between the infants, whose mothers are randomized to a TDF-containing triple ARV regimen vs. a CBV-containing triple ARV regimen during pregnancy, assessed as CrCl, Dpyr and bone mineral content (BMC) of both whole body (WB) and lumbar spine (LS) at delivery.

PP exposure

4. To compare the renal and bone toxicity between infants randomized to a maternal TDF-containing triple ARV regimen vs. infant NVP (no maternal ARVs) during breastfeeding, assessed as CrCl, Dpyr and LS BMC at 26 weeks of life.

AP and PP exposures

5. To compare fetal/infant somatic growth by TDF exposure status during pregnancy measured at delivery and during breastfeeding measured at 26 weeks of life, assessed as length-for-age Z-score, weight-for-age Z-score, and head-circumference Z-score, respectively.

2.2 Secondary Objectives

<u>Maternal</u>:

AP exposure

1. To compare the renal and bone toxicity between the groups randomized to a TDF-containing triple ARV regimen vs. a CBV-containing triple regimen during pregnancy, assessed as: changes in CrCl and Dpyr from delivery to 6, 26, and 74 weeks postpartum; and hip BMD at delivery and changes in hip and LS BMD from delivery to 74 weeks postpartum
2. To compare the renal and bone toxicity between the groups randomized to a TDF-containing triple ARV regimen vs. short-course ZDV with single dose nevirapine (sdNVP) during pregnancy, assessed as: CrCl, Dpyr and hip and LS BMD at delivery; changes in CrCl and Dpyr from delivery to 6, 26, and 74 weeks postpartum; and changes in hip and LS BMD from delivery to 74 weeks postpartum.

PP exposure

3. To compare the renal and bone toxicity between the groups randomized to a maternal TDF-containing triple ARV regimen vs. infant NVP (no-triple ARV regimen), assessed as: changes in CrCl and Dpyr from delivery to 6 and 26 weeks postpartum and changes in hip BMD from delivery to 74 weeks postpartum.
4. To compare the mineral composition of breast milk from mothers taking TDF to that of breast milk from mothers not taking TDF.
5. To evaluate the relationship between women's hip and LS BMD change from baseline to 74 weeks postpartum and the duration of breastfeeding, and if that relationship differs by TDF exposure category.

AP and PP exposure

6. To compare the renal and bone toxicity by TDF exposure during pregnancy and breastfeeding, assessed as changes in CrCl and Dpyr (if available) at delivery and 6, 26, and 74 weeks postpartum and changes in hip and LS BMD from delivery to 74 weeks postpartum.

PK analyses

7. To estimate maternal TFV pharmacokinetic parameters and drug exposure among women who take TDF during pregnancy, delivery and postpartum and to correlate maternal TFV exposure with markers for renal and bone toxicity.
8. To characterize the kinetics of TFV transfer from maternal plasma to breast milk and estimate infant breast milk TFV dose.

Infant:

AP exposure

9. To compare the renal and bone toxicity between the groups whose mothers were randomized to a TDF-containing triple ARV regimen vs. a CBV-containing triple ARV regimen during pregnancy, assessed as changes in CrCl and Dpyr from delivery to 10, 26 and 74 weeks of life and in LS BMC from delivery to 26 weeks of life.
10. To describe the concentrations of hormonal growth factors in infants in relation to infant growth percentile and antepartum TDF exposure status at delivery, 10 and 74 weeks of life.
11. To compare the renal and bone toxicity between the infants whose mothers are randomized to a TDF-containing triple ARV regimen vs. short-course ZDV with sdNVP during pregnancy, assessed as: CrCl, Dpyr and BMC of both WB and LS at delivery; changes in CrCl and Dpyr from delivery to 10, 26 and 74 weeks of life; and changes in LS BMC from delivery to 26 weeks of life.

PP exposure

12. To compare the renal and bone toxicity between the groups randomized to a maternal TDF-containing triple ARV regimen vs. infant NVP (no-triple ARV regimen), assessed as changes in CrCl and Dpyr from delivery to 10 and 74 weeks of life.
13. To estimate infant TFV exposure from breast milk using the estimated infant breastmilk drug dose, measured infant plasma drug concentrations and population modeling.

AP and PP exposure

14. To compare the renal and bone toxicity by maternal TDF exposure during pregnancy and breastfeeding assessed as CrCl and Dpyr (if available) at delivery and weeks 10, 26, and 74 of life and LS BMC at delivery and week 26.

2.3 Exploratory Objectives

1. To evaluate the effects of maternal TDF on the prevalence of proximal renal tubulopathy in both mothers and infants as measured by the following: Serum creatinine (age-adjusted norms), tubular re-absorption of phosphate (serum and urine creatinine and phosphate), euglycemic glycosuria (glucosuria in presence of normal serum glucose), proteinuria, hypophosphatemia.

2. To evaluate the effects of maternal TDF on bone health measures in both mothers and infants as measured by the following: Rate of osteopenia (only collected if obtained as part of clinical care), bone fracture incidence; Urine N-terminal [NTX], urine NTX/creatinine ratio), and serum C-terminal telopeptides [s-CTx] (bone resorption markers); bone-specific alkaline phosphatase [BS ALP], osteocalcin (bone formation markers).

3. To examine the effect of HIV infection status (infants only), HIV quantitative RNA (mothers only), CD4+ lymphocyte percent/count (HIV-infected participants only), increased levels of proinflammatory cytokines (IL-1B, NF-alpha, IL-6, RANKL, OPG) on the association between maternal TDF use and bone, kidney and infant growth endpoints.

3.0 STUDY DESIGN

This is a nested, comparative study of bone, renal and growth outcomes in subjects randomized in 1077BF or 1077FF to receive a maternal TDF-containing prophylaxis regimen or no maternal TDF-containing prophylaxis regimen during pregnancy or during breastfeeding. The substudy is designed to

examine 1) the effects of antepartum TDF exposure on women and their infants (AP analyses) and 2) the effects of TDF exposure during breastfeeding on women and their infants (PP analyses). A total of approximately 875 mother infant pairs are expected to be enrolled in the substudy. Mothers and their infants will be followed for 74 weeks postpartum. The maternal TDF vs. no maternal TDF during pregnancy (or AP exposure) part of the substudy will include women and their infants enrolled in the antepartum component of 1077BF or 1077FF; enrollment into the AP part will occur within 2 weeks of enrollment/ randomization into 1077BAor 1077FA. The maternal TDF vs. no maternal TDF during breastfeeding (or PP exposure) part of the substudy will include mothers and infants enrolled in the postpartum component of 1077BF during breastfeeding excluding those receiving a TDF-containing triple ARV regimen while pregnant; enrollment into the PP part will occur at the same time as randomization into the postpartum component of 1077BF (6 to 14 days after delivery). Approximately 475 mother-infant pairs will be enrolled in the AP part of P1084s and 400 mother-infant pairs will be enrolled in the PP part of P1084s, most of whom would have DXA scans (The antepartum component of 1077BF and 1077FF is described in Section 2.0 of those protocols respectively; the postpartum component of 1077BF is described in Section 4.0 of that protocol).

4.0 SELECTION AND ENROLLMENT OF SUBJECTS

Eligible subjects enrolled in the antepartum component of 1077BF (1077BA) and 1077FF (1077FA) will be recruited for the AP part of this substudy. Eligible subjects enrolled in the post-partum component of 1077BF (1077BP) will be recruited for the PP part of this substudy. 1077BF and 1077FF will only be conducted at non-US sites. (Subject eligibility criteria and enrollment into 1077BA and 1077FA are described in Section 2.0 of each protocol; subject eligibility criteria and enrollment into 1077BP are described in Section 4.0 of the 1077BF protocol). Enrollment into P1084s will be limited to 1077BF and 1077FF African sites that have been approved as P1084s DXA sites.

Pregnant women enrolled in 1077BA or 1077FA (randomized to a triple ARV regimen or short course ZDV regimen for PMTCT during pregnancy) will be offered participation in the AP part of this substudy as mother-infant pairs. Women will be asked to provide consent for the substudy. Women who meet the eligibility criteria in Section 4.1 will ideally be enrolled prior to initiation of study drug; however, women can be enrolled up to the Week 2 Visit of 1077BA or 1077FA (i.e. within 21 days after 1077BA/1077FA study entry) and prior to labor. A sample of the Informed Consent Form can be found in Appendix IV; further information related to informed consent can be found in Section 10.1.

Mothers and their infants enrolled in 1077BP (randomized to a maternal TDF-containing triple ARV regimen vs. infant NVP for PMTCT during breastfeeding) will be offered participation in the PP part of this substudy. Women will be asked to provide consent for the substudy. Women and their infants who meet the eligibility criteria in Section 4.2 will be enrolled on the same day as their 1077BP enrollment, which may take place from 6 to 14 days postpartum. A sample of the Informed Consent Form can be found in Appendix IV; further information related to informed consent can be found in Section 10.1.

Note: Mother-infant pairs enrolled in the AP TDF exposure part of the substudy are excluded from enrolling into the PP part of the substudy. However, they will continue to be followed with their infants on P1084s after delivery through 74 weeks postpartum and contribute to all analyses of TDF exposure part during pregnancy (AP analysis). They will only contribute to the secondary analyses for the breastfeeding exposure part (PP analyses).

See Section 4.3 for the timing of enrollment and additional enrollment procedures.

4.1 Antepartum Part of Substudy (TDF Exposure during Pregnancy)

4.1.1 Inclusion Criteria

4.1.1.1 Mother-infant pair enrolled in 1077BA or 1077FA.

4.1.1.2 At a clinical site that has been approved as a P1084s DXA site.

4.1.1.3 Enrolled in the substudy up to the Week 2 Visit of 1077BA/1077FA (within 21 days after 1077BA/1077FA study entry) and prior to the start of labor.

4.1.1.4 Willing and able to provide written informed consent to participate in this substudy.

Note: Under protocol Version 1.0, enrollment in the Antepartum Part of the substudy was open to all sites participating in 1077BF or 1077FF (with and without DXA scanners) but limited to women co-infected with HBV (and their infants), because only women coinfected with HBV were eligible to receive a TDF-containing prophylaxis regimen in the Antepartum Component of the main studies (1077BA and 1077FA). Under protocol Version 2.0, all women enrolled in 1077BA and 1077FA are potentially eligible for the Antepartum Part of the substudy (with their infants) because all such women are eligible to receive a TDF-containing prophylaxis regimen in 1077BA and 1077FA; however, to maximize the availability of BMD data for analysis in the Antepartum Part of the substudy, under protocol Version 2.0, enrollment in the Antepartum Part is limited to DXA sites only.

4.2 Postpartum Part of Substudy (TDF Exposure during Breastfeeding)

(Note: this applies only to the new enrollment to P1084s, i.e., those who were not enrolled to P1084s while on the AP component)

4.2.1 Inclusion Criteria

4.2.1.1 Mother and their infant enrolled in 1077BP.

4.2.1.2 At a clinical site that has been approved as a P1084s DXA site.

4.2.1.3 Enrolled in the substudy within 6 to 14 days of delivery, on the same day as enrollment in 1077BP

4.2.1.4 Willing and able to provide written informed consent to participate in this substudy.

4.2.2 Exclusion Criteria

4.2.2.1 TDF exposure during pregnancy.
[NOTE: TDF use for up to 12 days beginning at labor allowed]

4.2.2.2 Enrolled in the AP part of P1084s.

4.3 Enrollment Procedures

Prior to implementation of this study, each site must have the protocol document and the consent form approved by the local Institutional Review Board (IRB)/Ethics Committee (EC). A Site Implementation Plan (SIP) will be required from each site participating in the study. The plan must be submitted to the Protocol Team for review and approval before protocol registration can occur.

Prior to implementation of this protocol, and any subsequent full version amendments, each site must have the protocol and the protocol informed consent form approved, as appropriate, by their local institutional review board (IRB)/ethics committee (EC) and any other applicable regulatory entity (RE). Upon receiving final approval, sites will submit all required protocol registration documents to the DAIDS Protocol Registration Office (DAIDS PRO) at the Regulatory Support Center (RSC). The DAIDS PRO will review the submitted protocol registration packet to ensure that all of the required documents have been received.

The site-specific informed consent form (ICF) will be reviewed and approved by the DAIDS PRO and sites will receive an Initial Registration Notification from the DAIDS PRO that indicates successful completion of the protocol registration process. A copy of the Initial Registration Notification should be retained in the site's regulatory files.

Upon receiving final IRB/EC and any other applicable RE approval(s) for an amendment, sites should implement the amendment immediately. Sites are required to submit an amendment registration packet to the DAIDS PRO at the RSC. The DAIDS PRO will review the submitted protocol registration packet to ensure that all the required documents have been received. The site-specific ICF WILL NOT be reviewed and approved by the DAIDS PRO and sites will receive an Amendment Registration Notification when the DAIDS PRO receives a complete registration packet. A copy of the Amendment Registration Notification should be retained in the site's regulatory files.

For additional information on the protocol registration process and specific documents required for initial and amendment registrations, refer to the current version of the DAIDS Protocol Registration Manual.

4.3.1 Timing of Antepartum Registration

Eligible mother-infant pairs enrolled in 1077BA or 1077FA must enroll into this substudy within 21 days after 1077BA/1077FA study entry and prior to the start of labor.

4.3.2 Timing of Postpartum Registration

Eligible women and their infants enrolled in 1077BP, not previously enrolled in P1084s, must enroll into this substudy immediately following 1077BP study entry, i.e., on the same day.

5.0 STUDY TREATMENT

Study treatment is described in the IMPAACT 1077BF and 1077FF versions of the PROMISE study. No treatment is included in this substudy.

6.0 SUBJECT MANAGEMENT

6.1 Toxicity Management

Toxicity management is described in Appendix II of the IMPAACT 1077BF and 1077FF protocols.

6.2 Subject Management

Women will follow the schedule of evaluations in APPENDIX I and will be followed for 74 weeks post-delivery. Infants will follow the schedule of evaluations in APPENDIX II and will also be followed for 74 weeks from birth.

Refer to the Manual of Procedures (MOP) for detailed instructions in obtaining blood, cord blood, breast milk and urine samples for this substudy. The MOP also contains specific instructions for preparing subjects for DXA scans, performing DXA scans, transmitting DXA results, and maintenance and quality assurance of DXA scanners.

6.3 Concomitant Medication Guidelines

6.3.1 Disallowed Medications

This substudy will use the same list of prohibited medications as the IMPAACT 1077BF and 1077FF versions of the PROMISE study in which the women and their infants are enrolled.

6.3.2 Precautionary Medications

This substudy will use the same list of precautionary medications as the IMPAACT 1077BF and 1077FF versions of PROMISE in which the women and their infants are enrolled.

6.4 Timing of Availability of Substudy Evaluation Results

The following individual participant results of substudy-related laboratory evaluations will be available in real-time: maternal and infant calcium, phosphorous, and creatinine and maternal creatinine clearance.

The following substudy evaluations will be batched and results will not be available in real-time: infant creatinine clearance, maternal and infant bone turnover markers, hormones, vitamins, cytokines, all pharmacokinetic data and breast milk analyses. Maternal and infant DXA scan results will be batched and read centrally approximately every 6 months. At the conclusion of the study, the central reading of the baseline and last set of DXA results for each woman receiving DXA through the substudy will be made available to the site. In addition, central readings of all DXA scan results – as the results become available – for women who experience a fracture during the study will be made available to the site. None of the other batched test results will be made available to the sites.

6.5 Criteria for Treatment Discontinuation

Decision about discontinuation of study treatment will be made as outlined in the IMPAACT 1077BF and 1077FF versions of the PROMISE study.

Women and their infants will continue to be followed on the substudy (off study drug/on study) and follow the substudy schedule of evaluations, even if the women and/or their infants discontinue PROMISE study treatment.

Women who become pregnant while on the substudy will not have DXA scans while they are pregnant but they may participate in all other substudy evaluations; infants of women who subsequently become pregnant during the study may continue to have DXA scans as scheduled.

6.6 Criteria for Study Discontinuation

Participants will be withdrawn from study participations for the following reasons:

- Request by the participant to withdraw.
- Request of the primary care provider if s/he thinks the study is no longer in the best interest of the participant, after consultation with the protocol team.
- Participant judged by the investigator to be a significant risk of failing to comply with the provisions of the protocol as to cause harm to self or seriously interfere with the validity of the study results, after consultation with the protocol team.
- At the discretion of the IMPAACT leadership, NIAID, NICHD, the Office for Human Research Protections (OHRP), the US FDA, the pharmaceutical supplier(s), an in-country national health or regulatory agency, or the IRB/EC.
- Imprisonment or involuntary confinement in a medical facility (e.g., for psychiatric illness or infectious disease).

7.0 EXPEDITED REPORTING OF SERIOUS ADVERSE EVENTS

Expedited adverse event (EAE) reporting will follow the guidance in the IMPAACT 1077BF and 1077FF versions of the PROMISE study, as no treatment is included in this substudy.

8.0 STATISTICAL CONSIDERATIONS

8.1 General Design Issues

This substudy aims to evaluate the safety of the TDF -containing triple ARV regimen vs. the non-TDF-containing triple ARV regimen and the non-triple ARV regimens in randomized comparisons using selected mother-infant pairs who participate in the AP and PP components (1077BA, 1077FA and 1077BP) of the PROMISE study. The primary objectives are to compare specific renal and bone markers (creatinine clearance, bone resorption, and BMD/BMC) and infant growth in 1) mother-infant pairs receiving a maternal TDF-containing triple ARV regimen vs. a maternal CBV-containing triple ARV regimen during the antepartum period (AP analysis), and 2) mother-infant pairs receiving a maternal TDF-containing triple ARV regimen vs. no maternal ARVs during breastfeeding (PP analysis). Mother-infant pairs who received short-course ZDV + sdNVP/TRV antepartum will serve as an additional comparison group for the secondary AP analyses. The primary time point for evaluation of the primary AP objectives will be delivery; for the PP analysis, the primary time points for evaluation will be 74 weeks after delivery for the mothers and 26 weeks after birth for the infants.

Note that patients randomized in the PP component (1077BP) to the TDF-based triple ARV regimen and who received the CBV-based triple ARV regimen during the AP component (1077BA) may be permitted to remain on the CBV-based regimen, rather than switching to the TDF-based regimen. These patients taking the CBV-based triple ARV regimen during the PP component would be excluded from the primary PP comparison of the substudy. The study team expects the number of these cases to be small, such that excluding these participants will have minimal impact on statistical power or potential bias. If the number of such exclusions were to be substantial, then subjects switching to the TDF-based triple ARV regimen would be compared with those remaining on the CBV-based triple ARV regimen with respect to baseline data at the time of PP randomization. This comparison would probe for biases, which could have implications for the interpretation of the primary results of the PP part of the substudy.

A key analysis issue is the extent to which triple ARV exposure during pregnancy varies as a function of when women are randomized to the PROMISE study: where a range from 14 weeks to 36 gestation weeks appears likely. This may impact both effect sizes and variability in outcome measures. The AP analysis will be stratified by gestational age at randomization in an attempt to minimize confounding due to differential TDF exposure times.

8.2 Outcome Measures

8.2.1 Primary Outcome Measures

Table 1 below shows the timeline when the primary outcomes are measured for women and infants enrolled in the Antepartum Part:

**Table 1**			
**Primary Outcomes**	**AP Entry (randomization)**	**(Delivery/birth, up to week 1)**	**26 Weeks**
**Women**			
Creatinine clearance (CrCl)[1]		X	
Bone resorption (Dpyr)		X	
Lumbar Spine BMD via DXA[2]		X	
**Infants**			
Creatinine clearance (CrCl)[3]		X	
Bone resorption (Dpyr)		X	
**Bone mineral content (BMC) via DXA[2]:**			
Lumbar Spine (LS)		X	
Whole Body (WB)		X	
Length-for-age Z score[4]		X	X

[1] Maternal creatinine clearance will be calculated using the Cockroft-Gault equation, which includes weight and age factors.
[2] All sites will use late-model Hologic equipment and software capable of adult and infant DXA scanning.
[3] Infant creatinine clearance will be calculated using the Schwartz equation, which includes length and age factors.
[4] Length will be determined by infantometer. Standing height will be determined by stadiometer.

Table 2 below shows the timeline when the primary outcomes are measured for women and infants enrolled in the Postpartum Part:

**Table 2**			
**Primary Outcomes**	**PP Entry (Delivery/birth, up to week 1)**	**26 Weeks**	**74 Weeks**
**Women**			
Creatinine clearance (CrCl)[1]	X		X
Bone resorption (Dpyr)			X
Lumbar Spine BMD via DXA[2]	X		X
**Infants**			
Creatinine clearance (CrCl)[3]		X	
Bone resorption (Dpyr)		X	
**Bone mineral content (BMC) via DXA[2]:**			
Lumbar Spine (LS)		X	
Length-for-age Z score[4]	X	X	

[1] Maternal creatinine clearance will be calculated using the Cockroft-Gault equation, which includes weight and age factors.
[2] All sites will use late-model Hologic equipment and software capable of adult and infant DXA scanning.
[3] Infant creatinine clearance will be calculated using the Schwartz equation, which includes length and age factors.
[4] Length will be determined by infantometer. Standing height will be determined by stadiometer.

8.2.2 Secondary Outcome Measures

<u>For women</u>:

- CrCl and Dpyr measured at 6, 26 and 74 weeks.
- BMD measured at delivery and change in hip BMD from delivery to 74 weeks.
- Mineral composition of breast milk at 1, 6, 26 and 74 weeks.

<u>For infants</u>:

- Creatinine clearance (CrCl) and bone resorption (Dpyr) measured at 10, 26 and 74 weeks.
- LS BMC measured at 26 weeks.
- Infant growth at birth, 10 weeks and 74 weeks.
- Concentration of hormonal growth factors for infants at birth, 10 weeks, 26 weeks and 74 weeks.

8.2.3 Exploratory Outcome Measures

- Serum creatinine (age-adjusted norms).
- Tubular re-absorption of phosphate (serum and urine creatinine and phosphate).
- Glucosuria in presence of normal serum glucose (presence/absence and quantitative).
- Proteinuria (presence/absence and quantitative).
- Hypophosphatemia (quantified).
- Fracture incidence (rare; not primary).
- Rate of osteopenia (only collected if obtained as part of clinical care).
- Urine N-terminal [NTX]; serum C-terminal telopeptides [s-CTx]; urine NTX/creatinine ratio).

- Bone formation (increased BS ALP; increased osteocalcin).
- Cofactors (for adjusted analyses):
  - HIV infection status (infants only).
  - HIV quantitative RNA (mothers only).
  - Hepatitis B status (mothers only).
  - CD4+ lymphocyte percent/count (HIV-infected participants only).
  - 25-OH Vitamin D (vitamin D store), Parathyroid hormone (PTH).
  - Increased levels of proinflammatory cytokines (IL-1B, NF-alpha, IL-6, RANKL, OPG).
  - Gestational age at birth (infants only).
  - Achievement of weight-bearing motor milestones (infants only).
  - Nutritional intake – calcium, vitamin D, calories (mothers only).
  - Activity level (mothers only).
  - Cigarette and alcohol use (mothers only).

8.3 Randomization and Stratification

The substudy will use the AP randomization of the PROMISE protocol; triple ARV regimens (CBV/LPV-RTV or TDF/LPV-RTV) vs. ZDV + sdNVP + TRV tail and the PP randomization of the PROMISE protocol; a maternal triple ARV prophylaxis regimen (TDF/LPV-RTV) vs. infant NVP. No additional randomization will be done for the substudy.

8.4 Sample Size and Accrual

Due to cost concerns, enrollment into P1084s will be limited to 875 mother-infant pairs, with 475 pairs to be enrolled into P1084s at 1077AP randomization and 400 pairs at 1077BP randomization.

In version 1.0 of the P1084s protocol, only HBV+ pregnant women were eligible to be randomized to a TDF-containing regimen in the Antepartum Components of the main study (1077BA and 1077FA). Thus, enrollment into P1084s at Antepartum Component randomization was restricted to those HBV+ pregnant women. Under the current version of the main protocols (1077BA and 1077FA), all pregnant women will be eligible for randomization to a TDF-containing regimen; thus the sample in the Antepartum Part of P1084s will not be restricted to HBV+ women. In order to maximize the collection of DXA scan data, enrollment into the Antepartum Part of P1084s will be restricted to those African clinical sites that have been approved as P1084s DXA sites. The AP analyses will only include subjects who were enrolled into the Antepartum Part of P1084s.

Under the current version of the P1084s protocol, 400 mother-infant pairs will be enrolled into the Postpartum Part of P1084s from 1077BP, with enrollment restricted to African clinical sites that have been approved as P1084s DXA sites. For some analyses assessing the post-delivery effects of TDF, it will be appropriate to combine data from these 400 1077BP mother-infant pairs and those mother-infant pairs enrolled into the Antepartum Part of P1084s who were not randomized to a TDF-containing regimen in the main study but who were subsequently randomized to an ARV regimen in 1077BP. This will increase the potential sample sizes for some analyses.

Tables 3 and 4 illustrate the effect sizes which can be detected with 80% power with the sample sizes anticipated to be available for the various maternal and infant primary endpoints, respectively, using assumptions about the expected variability in the outcome measures and a

two-sided Student t-test with a type I error rate of 5%, without adjustment for multiple comparisons (considered as secondary analyses). The calculations use the evaluable sample size without adjusting for loss to follow-up (LFU), non-evaluable results/DXA scans, or the percentage of eligible subjects who would agree to enroll in the substudy. The references supporting the assumptions on which these calculations are based are cited in these tables. Note that there were cases in which the literature did not contain perfect matches for the analyses. Here we used the closest estimates available and attempted to adjust the assumptions in a conservative direction.

While there are not clear guidelines for establishing the difference in LS BMD (younger women), LS BMC or WB BMC (infants) that would be considered clinically relevant, most experts believe that a difference as small as 5-7 % LS BMD (women), LS BMC (infant) and WB BMC (infant) or a difference of 0.5-0.75 Standard Deviation (SD), would be of enough concern to potentially influence decision-making about therapy. Thus, the goal will be to ensure the available sample size to detect a LS BMD (women), LS BMC or WB BMC (infants) difference as small as 5-7 % and/or 0.5 SD, where possible, with adequate power of 80%.

Table 3 Mothers Sample Sizes and Effect Sizes Required for 80% Statistical Power

Outcome measures	Time	References	Assumptions		Sample Size for 80% Power
CrCl	Delivery	Gallant et al.:<br>se=3.1, n=555<br>SD=3.1*sqrt(555)=73<br>Mean GFR (pregnant African women) assumed to be 120	Effect size: 25% of mean GFR<br>(120 X .25 = 30)		N per group<br>94
			Effect size (SD): 30(73)		
	Change from delivery to 26 weeks	Gilead et al.; Gallant et al.<br>SD = 40<br>Mean GFR (African women) assumed to be 100 at 6 months after delivery	SD=70 (to adjust for greater variability, relative to Gilead and Gallant designs)<br>Effect size to be 25% of 6-month mean GFR<br>(100 X .25 = 25)		N per group<br>124
			Effect size (SD): 25 (70)		
BMD (lumbar spine) g/cm^2	Delivery	Black et al.<br>mean=0.8<br>SD=0.14	Difference to be detected	Effect size (SD)	N per group
			4% (of 0.8)	0.032 (0.14)	301
			5%	0.04	193
			6%	0.048	134
			7%	0.056	99
			8%	0.064	76
			0.5*SD	0.07	63
			0.75*SD	0.105	28
			1*SD	0.14	16
BMD<br>(% lumbar spine change)	Change from delivery to 26 weeks	From VOICE study<br>SD=3.5%-5.5 %<br>(use 5.5%)	Effect size (SD): 2% (5.5%)		N per group<br>119
Bone resorption<br>Dpyr<br>(pmol/umol creat)<br>(Deoxypyridinoine)	Delivery	P Szulc et al.<br>Mean=18<br>SD=5 (used SD=10; pregnancy will increase variation)	Effect size: 20% of mean Dpyr<br>(18 X .20 = 3.6)<br>SD=10		N per group<br>123
			Effect size (SD): 3.6 (10)		
	At 26 weeks	P Szulc et al.<br>Mean=18<br>SD=5	Effect size: 10% of mean Dpyr<br>(18 X .10 = 1.8)<br>SD=5		N per group<br>123
			Effect size (SD): 1.8 (5)		

Table 4 Infant Sample Sizes and Effect Sizes Required for 80% Power

Outcome Measures	Time	References	Assumptions		Sample Size for 80% Power
Whole Body BMC<br>WB BMC (g)	Birth	Picaud et al.<br>Mean=26; SD=3.6	Diff to be detected	Effect size (SD)	N per group
			4% (of 26)	1.04 (3.6)	189
			5%	1.3	121
			6%	1.56	84
			7%	1.82	62
			8%	2.08	48
			0.5*SD	1.8	63
			0.75*SD	2.7	28
			1*SD	3.6	16
Lumbar Spine 1-4<br>BMC (g)	Birth	Weiler et al.<br>Mean=2.4, SD=0.5<br>(White)	Assumptions		
			Diff to be detected	Effect size (SD)	N per group
			4% (of 2.4)	0.096 (0.5)	426
			5%	0.120	273
			6%	0.144	190
			7%	0.168	140
			8%	0.192	107
			0.5*SD	0.250	63
			0.75*SD	0.375	28
			1*SD	0.50	16
	At 26 wks	Heidi Kalkwarf's data<br>Mean=3.8, SD=1.00	Assumptions		
			Diff to be detected	Effect size (SD)	N per group
			4% of mean (=3.8)	0.152 (1.0)	680
			5%	0.190	435
			6%	0.228	302
			7%	0.266	222
			8%	0.304	170
			0.5SD	0.50	63
			0.75SD	0.75	28
			1.0SD	1.00	16
CrCl<br>(mL/min/1.73m^2)	Birth	Schwartz et al.<br>Baseline mean=50<br>se=5.8, n=9<br>sd=5.8*sqrt(9)=~18	Effect size: 15% of mean<br>(50 X .15 = 7.5)<br>Effect size (SD): 7.5		N per group<br>92
	At 26 weeks	Schwartz et al.<br>Mean=88<br>Se=12, n=11<br>sd=12*sqrt(11)=~40	Effect size: 15% of mean<br>(88 X .15 = 13.2)<br>Effect size (SD): 13.2		N per group<br>145
Dpyr<br>(pmol/umol creat)<br>(Deoxypyridinoine)	Birth	P Szulc et al.<br>Mean=120 ; SD=~35<br>for 3-year-olds	Effect size: used 40<br>SD= 50<br>Effect size (SD): 40 (50)		N per group<br>26
	At 26 weeks	P Szulc et al.<br>Mean=120 ; SD=~35<br>for 3-year-olds	Effect size: 15% of mean<br>(120 X .15 = 18)<br>SD: used 50<br>Effect size (SD): 18 (50)		N per group<br>123
Length for Age	Birth	WHO Charts<br>Mean=~50 cm ; SD=2 cm	Effect size to be a<br>Z-score of .4 (.8 cm)<br>Effect size (SD): 8 cm (2 cm)		N per group<br>100
		WHO Charts<br>Mean=~66 cm ; SD=2 cm	Effect size to be a<br>Z-score of .4 (.8 cm)<br>Effect size (SD) .8cm (2 cm)		N per group<br>100

8.5 Monitoring

Routine monitoring, which will be performed by the substudy team (or a subset of the study team), will include the following: accrual, study status/progress, data and specimen timeliness and completeness and adverse events. A monitoring plan with specific details concerning the content and scheduling of these reports will be disseminated in a separate document before the study opens to accrual.

The interim or NIAID-sponsored Data and Safety Monitoring Board (DSMB) monitoring for this substudy will be integrated with those of the main components of the PROMISE study (1077BA, 1077FA and 1077BP). Since much of the primary safety data for this substudy (e.g., maternal creatinine and creatinine clearance, all fractures and all pathologic fractures) are being monitored by the main PROMISE study and will be assessed at each interim evaluation of the respective PROMISE (1077BA, 1077FA and1077BP) components, a summary of the available safety data for the substudy (i.e. maternal and infant calcium, phosphorous, creatinine, maternal creatinine clearance, and infant growth measurement, collapsed over study arms) will be included in the Antepartum or Postpartum component DSMB report prepared for each interim review and the summary will be provided to the substudy team for review.

Data collected only on subjects in this substudy for the purpose of measuring bone toxicity include: LS BMD for women, and LS BMC and WB BMC for infants. These data will come from DXA scans, which will be read in batch. At the time each main PROMISE DSMB report (for 1077BA, 1077FA and 1077BP) is presented, it will include descriptive analyses of DXA data that becomes available from subjects participating in this substudy. These data will be broken down by treatment groups (with treatments masked) in the closed DSMB report and will allow the DSMB to monitor whether there are important differences in bone density that may be attributable to treatment assignment in this substudy.

8.6 Analyses

8.6.1 Primary Analysis

For the primary AP analysis, Student t-tests (two-sided) will be used to compare the TDF based triple ARV regimen group vs. the CBV based triple ARV regimen group at the time of delivery with respect to the primary outcome measures. Pairwise comparisons of each antepartum triple ARV regimen group and the antepartum short-course ZDV group will be performed as secondary analyses. Similar methods will be used for the PP analyses to compare the TDF based triple ARV regimen group vs. no triple ARV regimen group at 26 weeks postpartum for the infants and at 74 weeks postpartum for the mothers. If there are cases where outcome variables do not meet normality assumptions, the Wilcoxon Rank-Sum test will be used. Descriptive analyses will also be performed to examine whether the effects of the TDF based triple ARV regimen for the AP analysis are relatively consistent across revisions of the protocol.

8.6.2 Secondary Analysis

Similar methods will be used to compare these groups with respect to the secondary endpoints described in Section 8.2.2. One analysis would be to use the infant birth DXA results as a covariate in a regression model comparing PP treatment arms with respect to infant DXA results at week 26 of life. Further secondary analyses will use regression models to control for potential confounders. These models are designed to examine

whether statistically significant treatment effects observed in univariate analyses remain significant after controlling for potential confounders. They will also test whether there are interaction effects such that effects observed during postpartum period upon the treatment received during the antepartum period, although the power to detect such interactions would be extremely limited.

It is also possible that initiation of a triple ARV regimen (with or without TDF) could affect bone and renal parameters. To examine whether some safety results reflect the onset of a triple ARV regimen, rather than the specific effects of TDF, a subgroup analysis will compare PP a maternal TDF based triple ARV regimen vs. a non-triple ARV regimen separately among those who had been randomized in antepartum to a TDF-based triple ARV regimen vs. those who were on a CBV-based triple ARV regimen or short-course ZDV + sdNVP + TRV tail.

9.0 CLINCAL PHARMACOLOGY PLAN

9.1 Rationale

P1084s, a nested, comparative substudy of 1077BF and 1077FF, will monitor mothers and their infants for bone, kidney and infant growth outcomes. The pharmacology component of P1084s will describe maternal blood, cord blood, breast milk and infant blood concentrations of TFV among women who take TDF during pregnancy, delivery and lactation and their infants and will attempt to correlate maternal and infant TFV exposure with measures of TFV toxicity.

Whenever any drug is used in a lactating woman, a major consideration has to be the kinetics of drug transfer into the infant via breast milk. Previous studies with ZDV, NVP, nelfinavir and 3TC have shown different patterns of transfer for each drug. Transfer of ZDV and nelfinavir from mother to infant is negligible (76). In contrast, transfer of 3TC, EFV and NVP from mother to infant via breast milk results in clinically significant but subtherapeutic concentrations (26, 77). These concentrations are too low to result in complete viral suppression, making them likely to lead to the development of NVP and 3TC viral resistance in infected infants (78). There is currently no data available describing mother to child breast milk drug transfer of TFV. This substudy offers a unique opportunity to generate these data and to correlate maternal and infant TFV exposure with renal and bone outcomes.

9.2 Pharmacology Objectives

9.2.1 To estimate maternal TFV pharmacokinetic parameters and drug exposure among women who take TDF during pregnancy, delivery and postpartum and to correlate maternal TFV exposure with markers for renal and bone toxicity.

9.2.2 To characterize the kinetics of TFV transfer from maternal plasma to breast milk and estimate infant breast milk TFV dose.

9.2.3 To estimate infant TFV exposure from breast milk using the estimated infant breast milk drug dose, measured infant plasma drug concentrations and population modeling.

9.3 Study Design, Modeling and Data Analysis

9.3.1 Study Design – Sampling and Assay Strategy

9.3.1.1 Maternal exposure component – Stored samples of maternal plasma collected at the following timepoints: 1, 6, 26 and 74 weeks from 150 women will be assayed for TFV concentration.

9.3.1.2 Breast milk transfer component – Stored samples of maternal plasma, cord blood (labor and delivery visit only), breast milk and infant blood (plasma or dried blood spot) from 50 breast feeding mother-infant pairs receiving TDF collected at all substudy visits and at the week 14 visit of the parent 1077BF study will be assayed for TFV concentration. Breast milk and infant samples will be assayed only if the infant is continuing to breast feed at the time of sample collection.

9.3.1.3 Assays for TFV will be assigned to one of the IMPAACT Pharmacology Laboratories, depending on laboratory expertise and work load. Demographic and clinical data will be compiled from the 1077BF and P1084s data bases.

9.3.2 Modeling and Data Analysis

Objective 1: *To estimate maternal TFV pharmacokinetic parameters and drug exposure among women who take TDF during pregnancy, delivery and postpartum and to correlate maternal TFV exposure with markers for renal and bone toxicity.* A two-stage approach will be used. First, descriptive pharmacologic analyses will be performed by generating summed maternal TFV concentration time plots. These plots will be compared graphically with existing data describing the distribution of TFV concentrations over time in non-pregnant adults, using reference ranges derived from postpartum pharmacokinetic results from P1026s. Monte Carlo simulations will be used to construct confidence interval bands for expected concentrations encompassing the following intervals: $< 10^{th}$, 10th-$< 25^{th}$, 25th-$< 50^{th}$, 50th-$< 75^{th}$, 75th-$< 90^{th}$ and $> 90^{th}$ percentiles. The measured maternal plasma TFV concentrations in the nursing P1084s and non-nursing P1084s women will be compared to those postpartum from P1084s. The percentile band will be determined for each P1084s maternal pharmacokinetic sample. These comparisons will provide an initial indication of whether differences exist in TFV exposure between the nursing and non-nursing P1084s mothers and the reference populations. The second stage of the analysis will use pharmacokinetic modeling techniques to provide estimates of TFV pharmacokinetic parameters and drug exposure for the P0184s women and to make statistical comparisons between the women and the reference population. Among the modeling techniques used will be least squares mixed effects modeling. In this approach, pharmacokinetic parameters describing TFV kinetics in maternal blood, including intra- and inter-patient variability, will be estimated using nonlinear hierarchical models and an extended least squares method using the software program NONMEM. The data will be nested with the maternal pharmacokinetic data from P1026s. Other components of the P1084s substudy involve describing clinical markers for maternal and infant renal and bone toxicity. We will perform exploratory analyses looking for relationships between these markers and maternal TFV exposure parameters. With a sample size of 200 women (150 as per section 9.3.1.1 and 50 as per

section 9.3.1.2), we hope to be able to evaluate hypotheses relating maternal TFV exposure with maternal and infant renal and bone toxicity.

Objective 2: *To characterize the kinetics of TFV transfer from maternal plasma to breast milk and estimate infant breast milk TFV dose.* A similar two-stage approach will be used to analyze the breast milk data. First, descriptive pharmacologic analyses will be performed comparing absolute breast milk TFV concentrations and milk to plasma ratios to temporal and demographic variables, including time from maternal dosing, time from last feeding and time since delivery. The second stage will use pharmacokinetic parameters determined in Objective 1 and a similar modeling approach to the breast milk TFV concentration data. The kinetics of TFV transfer from plasma to breast milk and an estimated TFV dose delivered to the infant in breast milk will be modeled. The model will incorporate breast milk as a pharmacokinetic compartment and model the plasma and breast milk TFV concentrations concomitantly using a semi-physiologic approach. Other components of the P1084s substudy will describe mineral composition of maternal breast milk. Exploratory analyses will look for relationships between breast milk mineral composition and maternal blood and breast milk TFV exposure parameters. While the sample size may be too small for definitive conclusions, at a minimum, preliminary hypotheses, which relate maternal TFV exposure and breast milk mineral composition, can be developed.

Objective 3: *To estimate infant TFV exposure from breast milk using the estimated infant breast milk drug dose from Objective 2, measured infant plasma drug concentrations and population modeling.* Infant TFV exposure will be estimated using modeling techniques. The breast milk drug doses described above and an assumed consumption of 150 mL/kg/day of breast milk will initially be used for infant drug input (79). The infant drug exposure will be modeled as a function of maternal plasma pharmacokinetics, time of maternal dosing, timing of infant feedings and maturational state of the infant. Analyses performed for Objectives 1 and 2 will provide will the maternal information necessary for the assessment of infant pharmacokinetics if appreciable infant TFV concentrations are found. Models of maternal plasma and breast milk kinetics will be linked and modeled simultaneously with the infant PK models. The infant models may be nested with existing developmental models of drug disposition where data exist or from more general models of drug metabolizing enzyme ontogeny (80). Simultaneous modeling should provide a more accurate description of the pharmacokinetic parameter space since mothers with higher TFV concentrations are likely to give their infants larger TFV doses through breast milk. The final models will be used to estimate the range of TFV exposures likely to be seen in BF infants over the first 6 months of life. Monte Carlo simulations will be performed with 2,000 individuals at each visit age using the Simulation subroutine of NONMEM to provide the infant TFV exposure pattern expected during the first 6 months of life through BF.

10.0 HUMAN SUBJECTS

10.1 Institutional Review Board and Informed Consent

This protocol, the informed consent document (APPENDIX IV), and any subsequent modifications must be reviewed and approved by the IRB or EC responsible for oversight of the substudy. Written informed consent must be obtained from the subject, in this substudy the enrolled mother with give consent for herself and her infant to participate. The informed consent

will describe the purpose of the substudy, the procedures to be followed and the risks and benefits of participation. A copy of the consent form will be offered to the subject.

Each site which receives US HHS funding and follows the United States Code of Federal Regulations Title 45-Public Welfare, Part 46-Protection of Human Subjects (also known as the Common Rule) should have on record at the site a plan that detects and addresses any change in guardianship occurring in pediatric subjects and determines when a study subject must have a consent process which involves a legally authorized representative (LAR) other than a family member with guardianship. The plan will include how the site determines when a LAR is initially or no longer needed and how frequently the LAR re-signs the consent. The plan should follow all IRB/EC, local, state, national and/or host country guidelines. Confirmation of such a plan at a site should be submitted with protocol registration materials. Should the mother of an enrolled infant die or no longer be available for any other reason no further substudy-specific evaluations or assessments can be performed until consent for the infant's continued participation in the substudy is obtained from a legally authorized individual, as defined locally. However, sites should continue to provide care for the infant as needed and appropriate (outside of the study).

10.2 Subject Confidentiality

All laboratory specimens, evaluation forms, reports, and other records that are transferred or transmitted off-site for processing will be identified only by a coded number to maintain subject confidentiality. All records will be kept in a secured area with access limited to authorized personnel only. All computer entry and networking programs will be performed with coded numbers only. The use of participant identifiers on study records must comply with the DAIDS policies. Clinical information will not be released without written permission of the subject, except as necessary for monitoring by the US FDA, the Office for Human Research Protections (OHRP), the study sponsors (NIAID and NICHD) or their authorized agents, representatives or agents of the IMPAACT leadership (e.g., staff from the operations center, data management center and network lab), the IRBs/ECs, local regulatory authorities or the pharmaceutical co-sponsors.

10.3 Study Discontinuation

The study may be discontinued at any time by the IMPAACT leadership, NIAID, NICHD, the Office for Human Research Protections (OHRP), the US FDA, the pharmaceutical co-sponsors, an in-country national health and/or regulatory agencies and/or the IRBs/ECs as part of their duties to ensure that research subjects are protected.

11.0 PUBLICATION OF RESEARCH FINDINGS

Publication of the results of this trial will be governed by IMPAACT policies. Any presentation, abstract or manuscript will be made available for review by the pharmaceutical sponsors prior to submission.

12.0 BIOHAZARD CONTAINMENT

As the transmission of HIV and other bloodborne pathogens can occur through contact with contaminated needles, blood, and blood products, appropriate blood and secretion precautions will be employed by all personnel in the drawing of blood and shipping and handling of all specimens for this study, as currently recommended by the Centers for Disease Control and Prevention.

All infectious specimens will be sent using the ISS-1 SAF-T-PAK mandated by the International Air Transport Association Dangerous Goods Regulations-Packing Instruction 602. Refer to individual carrier guidelines (e.g., Federal Express or Airborne) for specific instructions.

13.0 REFERENCES

1. Panel on Antiretroviral Guidelines for Adults and Adolescents. Guidelines for the use of antiretroviral agents in HIV-1-infected adults and adolescents. Bethesda (MD): Department of Health and Human Services (DHHS); 2008 Nov 3. 139 p.
2. Jullien V, Treluyer J, Rey E, Jaffray P, Krivine A, Moachon L, et al. Population pharmacokinetics of tenofovir in human immunodeficiency virus-infected patients taking highly active antiretroviral therapy. Antimicrob Agents Chemother 2005;49(8):3361-6.
3. Van Rompay K, Durand-Gasselin L, Brignolo L, Ray A, Abel K, Cihlar T, et al. Chronic administration of tenofovir to rhesus macaques from infancy through adulthood and pregnancy: summary of pharmacokinetics, biological and virological effects. Antimicrob Agents Chemother; 2008;52(9): 3144-60.
4. Van Rompay KK, Brignolo LL, Meyer DJ, Jerome C, Tarara R, Spinner A, et al. Biological effects of short-term or prolonged administration of 9-[2-(phosphonomethoxy)propyl]adenine (tenofovir) to newborn and infant rhesus macaques. Antimicrob Agents Chemother 2004 May;48(5):1469-87.
5. Izzedine H, Isnard-Bagnis C, Hulot JS, Vittecoq D, Cheng A, Jais CK, et al. Renal safety of tenofovir in HIV treatment-experienced patients. AIDS 2004 Apr 30;18(7):1074-6.
6. Gallant JE, Deresinski S. Tenofovir disoproxil fumarate. Clin Infect Dis 2003 Oct 1;37(7):944-50.
7. Padilla S, Gutierrez F, Masia M, Canovas V, Orozco C. Low frequency of renal function impairment during one-year of therapy with tenofovir-containing regimens in the real-world: a case-control study. AIDS Patient Care STDs 2005 Jul;19(7):421-4.
8. Lanzafame M, Lattuada E, Rapagna F, Gottardi M, Vento S. Tenofovir-associated kidney diseases and interactions between tenofovir and other antiretrovirals. Clin Infect Dis 2006 Jun 1;42(11):1656-7; author reply 8.
9. Scott JD, Wolfe PR, Bolan RK, Guyer B. Serious renal impairment occurs rarely with use of tenofovir DF. HIV Clin Trials 2006 Mar-Apr;7(2):55-8.
10. Gallant JE, DeJesus E, Arribas JR, Pozniak AL, Gazzard B, Campo RE, et al. Tenofovir DF, emtricitabine, and efavirenz vs. zidovudine, lamivudine, and efavirenz for HIV. N Engl J Med 2006 Jan 19;354(3):251-60.
11. Antoniou T, Raboud J, Chirhin S, Yoong D, Govan V, Gough K, et al. Incidence of and risk factors for tenofovir-induced nephrotoxicity: a retrospective cohort study. HIV Med 2005 Jul;6(4):284-90.
12. Gallant JE, Winston JA, DeJesus E, Pozniak AL, Chen SS, Cheng AK, et al. The 3-year renal safety of a tenofovir disoproxil fumarate vs. a thymidine analogue-containing regimen in antiretroviral-naive patients. AIDS 2008 Oct 18;22(16):2155-63.
13. Murphy MD, O'Hearn M, Chou S. Fatal lactic acidosis and acute renal failure after addition of tenofovir to an antiretroviral regimen containing didanosine. Clin Infect Dis 2003 Apr 15;36(8):1082-5.
14. Karras A, Lafaurie M, Furco A, Bourgarit A, Droz D, Sereni D, et al. Tenofovir-related nephrotoxicity in human immunodeficiency virus-infected patients: three cases of renal failure, Fanconi syndrome, and nephrogenic diabetes insipidus. Clin Infect Dis 2003 Apr 15;36(8):1070-3.
15. Barrios A, Garcia-Benayas T, Gonzalez-Lahoz J, Soriano V. Tenofovir-related nephrotoxicity in HIV-infected patients. AIDS 2004 Apr 9;18(6):960-3.
16. Gaspar G, Monereo A, Garcia-Reyne A, de Guzman M. Fanconi syndrome and acute renal failure in a patient treated with tenofovir: a call for caution. AIDS 2004 Jan 23;18(2):351-2.
17. James CW, Steinhaus MC, Szabo S, Dressier RM. Tenofovir-related nephrotoxicity: case report and review of the literature. Pharmacotherapy 2004 Mar;24(3):415-8.

18. Peyriere H, Reynes J, Rouanet I, Daniel N, de Boever CM, Mauboussin JM, et al. Renal tubular dysfunction associated with tenofovir therapy: report of 7 cases. J Acquir Immune Defic Syndr 2004 Mar 1;35(3):269-73.
19. Labarga P, Barreiro P, Martin-Carbonero L, Rodriguez-Novoa S, Solera C, Medrano J, et al. Kidney tubular abnormalities in the absence of impaired glomerular function in HIV patients treated with tenofovir. AIDS 2009 Mar 27;23(6):689-96.
20. Nurutdinova D, Onen NF, Hayes E, Mondy K, Overton ET. Adverse effects of tenofovir use in HIV-infected pregnant women and their infants. Ann Pharmacother 2008 Nov;42(11):1581-5.
21. Hussain S, Khayat A, Tolaymat A, Rathore MH. Nephrotoxicity in a child with perinatal HIV on tenofovir, didanosine and lopinavir/ritonavir. Pediatr Nephrol 2006 Jul;21(7):1034-6.
22. Wood SM, Shah SS, Steenhoff AP, Meyers KE, Kaplan BS, Rutstein RM. Tenofovir-associated nephrotoxicity in two HIV-infected adolescent males. AIDS Patient Care STDs 2009 Jan;23(1):1-4.
23. Andiman W, Chernoff M, Mitchell C, Oleske J, Seage III G, Tappenden J, et al. Occurrence of Renal Diseases and Laboratory Abnormalities among HIV-infected Children Enrolled in PACTG 219/219C. 13th Conference on Retroviruses and Opportunistic Infections; Denver, CO 2006.
24. Riordan A, Judd A, Boyd K, Cliff D, Doerholt K, Lyall H, et al. Tenofovir use in human immunodeficiency virus-1-infected children in the United kingdom and Ireland. Pediatr Infect Dis J 2009 Mar;28(3):204-9.
25. Flynn P, Mirochnick M, Shapiro D, al e, editors. Single-dose Tenofovir Disoproxil Fumerate with and without Emtricatrabine in HIV-1 infected pregant women and their infants: Pharmacokinetics and Safety [abstract no. 939]. 16th Conference on Retroviruses and Opportunistic Infections; 2009; Canada.
26. Mirochnick M, Thomas T, Capparelli E, Zeh C, Holland D, Masaba R, et al. Antiretroviral Concentrations in Breast-Feeding Infants of Mothers Receiving Highly Active Antiretroviral Therapy. Antimicrob Agents Chemother 2009;53(3):1170.
27. Wood CE, Cousins R, Zhang D, Keller-Wood M. Ontogeny of expression of organic anion transporters 1 and 3 in ovine fetal and neonatal kidney. Exp Biol Med (Maywood) 2005 Oct;230(9):668-73.
28. Robertson J, Shilkofski N. The Harriet Lane handbook: a manual for pediatric house officers: Mosby; 2005.
29. Bartlettt JG, Gallant JE. Medical Management of HIV Infection. Baltimore, MD: Johns Hopkins Medicine, Health Publishing Business Group; 2007.
30. Stohr W, Walker AS, Munderi P, Tugume S, Gilks CF, Darbyshire JH, et al. Estimating glomerular filtration rate in HIV-infected adults in Africa: comparison of Cockcroft-Gault and Modification of Diet in Renal Disease formulae. Antivir Ther 2008;13(6):761-70.
31. Castillo AB, Tarantal AF, Watnik MR, Martin RB. Tenofovir treatment at 30 mg/kg/day can inhibit cortical bone mineralization in growing rhesus monkeys (Macaca mulatta). J Orthop Res 2002 Nov;20(6):1185-9.
32. Triant VA, Brown TT, Lee H, Grinspoon SK. Fracture prevalence among human immunodeficiency virus (HIV)-infected versus non-HIV-infected patients in a large U.S. healthcare system. J Clin Endocrinol Metab 2008 Sep;93(9):3499-504.
33. Bedimo R, Maalouf NM, Zhang S, Drechsler H, Tebas P. Osteoporotic fracture risk associated with cumulative exposure to tenofovir and other antiretroviral agents. AIDS. 2012 Apr 24;26(7):825-31.
34. Mundy LM, Youk AO, McComsey GA, Bowlin SJ. Overall benefit of antiretroviral treatment on the risk of fracture in HIV: nested case control analysis in a health-insured population. AIDS. 2012 Feb 1. [Epub ahead of print]
35. O'Brien KO, Razavi M, Henderson RA, Caballero B, Ellis KJ. Bone mineral content in girls perinatally infected with HIV. Am J Clin Nutr 2001 Apr;73(4):821-6.

36. Mora S, Zamproni I, Beccio S, Bianchi R, Giacomet V, Vigano A. Longitudinal changes of bone mineral density and metabolism in antiretroviral-treated human immunodeficiency virus-infected children. J Clin Endocrinol Metab 2004 Jan;89(1):24-8.
37. Arpadi SM, Horlick M, Thornton J, Cuff PA, Wang J, Kotler DP. Bone mineral content is lower in prepubertal HIV-infected children. J Acquir Immune Defic Syndr 2002 Apr 15;29(5):450-4.
38. Mora S, Sala N, Bricalli D, Zuin G, Chiumello G, Vigano A. Bone mineral loss through increased bone turnover in HIV-infected children treated with highly active antiretroviral therapy. AIDS 2001 Sep 28;15(14):1823-9.
39. Jacobson DL, Spiegelman D, Duggan C, Weinberg GA, Bechard L, Furuta L, et al. Predictors of bone mineral density in human immunodeficiency virus-1 infected children. J Pediatr Gastroenterol Nutr 2005 Sep;41(3):339-46.
40. Gallant J, Staszewski S, Pozniak A, DeJesus E, Suleiman J, Miller M, et al. Efficacy and Safety of Tenofovir DF vs Stavudine in Combination Therapy in Antiretroviral-Naive Patients A 3-Year Randomized Trial. JAMA2004;292(2):191-201.
41. Gafni RI, Hazra R, Reynolds JC, Maldarelli F, Tullio AN, DeCarlo E, et al. Tenofovir disoproxil fumarate and an optimized background regimen of antiretroviral agents as salvage therapy: impact on bone mineral density in HIV-infected children. Pediatrics 2006 Sep;118(3):e711-8.
42. Hazra R, Gafni RI, Maldarelli F, Balis FM, Tullio AN, DeCarlo E, et al. Tenofovir disoproxil fumarate and an optimized background regimen of antiretroviral agents as salvage therapy for pediatric HIV infection. Pediatrics 2005 Dec;116(6):e846-54.
43. Giacomet V, Mora S, Martelli L, Merlo M, Sciannamblo M, Vigano A. A 12-month treatment with tenofovir does not impair bone mineral accrual in HIV-infected children. J Acquir Immune Defic Syndr 2005 Dec 1;40(4):448-50.
44. Negra MD, de Carvalho AP, de Aquino MZ, da Silva MT, Pinto J, White K, Arterburn S, Liu YP, Enejosa JV, Cheng AK, Chuck SL, Rhee MS. A Randomized Studyof Tenofovir Disoproxil Fumarate in Treatment-Experienced Human Immunodeficiency Virus-1 Infected Adolescents. Pediatr Infect Dis J. 2012 Feb 1. [Epub ahead ofprint]
45. Siberry GK, Li H, Jacobson D; Pediatric AIDS Clinical Trials Group (PACTG) 219/219C Study. Fracture risk by HIV infection status in perinatally HIV-exposed children. AIDS Res Hum Retroviruses. 2012 Mar;28(3):247-50.
46. Nielsen-Saines K, Mirochnick M, Boechat MI, Kafulafula G, Kreitchmann R, Mwatha A, Pinto J, Swenson M, Sato P, Mofenson L. Bone Age and Mineral Density Assessments Using Plain Roentgenograms in Tenofovir (TDF) exposed infants in Malawi and Brazil Enrolled in HPTN 057. Pediatric Academic Societies Meeting, Vancouver, Canada, May, 2010.
47. Tarantal A, Castillo A, Ekert J, Bischofberger N, Martin R. Fetal and Maternal Outcome After Administration of Tenofovir to Gravid Rhesus Monkeys (Macaca mulatta). J Acquir Immune Defic Syndr 2002;29(3):207.
48. Sihem B, Hirt D, Odile L, Pannier E, et al. Pharmacology: Pregnancy-Related Effects on Tenofovir Pharmacokinetics: a Population Study with 186 Women, Antimicrob. Agents Chemother. February 2012; 56:857-862; published ahead of print 28 November 2011
49. Mirochnick M, Kafulafula G, Kreitchmann R, Pinto J, Parsons T, Nielsen-Saines K, Sato P, Mofenson L, Fowler MG. The pharmacokinetics (PK) of tenofovir disoproxil fumarate (TDF) after administration to HIV-1 infected pregnant women and their newborns. The 16th Conference on Retroviruses and Opportunistic Infections, Montreal, Canada, February, 2009.
50. Burchett S, Best B, Mirochnick M, Hu C, Capparelli E, Holland D, et al., editors. Tenofovir pharmacokinetics during pregnancy, at delivery and post partum [abstract no. 738b] 14th Conference on Retroviruses and Opportunistic Infections; 2007; Los Angeles, LA.
51. Griner R, Williams PL, Read JS, Seage GR 3rd, Crain M, Yogev R, Hazra R, Rich K; Pediatric HIV/AIDS Cohort Study. In utero and postnatal exposure to antiretrovirals among HIV-exposed but uninfected children in the United States. AIDS Patient Care STDS. 2011 Jul;25(7):385-94.

52. Siberry GK, Williams PL, Mendez H, Seage GR 3rd, Jacobson DL, Hazra R, Rich KC, Griner R, Tassiopoulos K, Kacanek D, Mofenson LM, Miller T, Dimeglio LA, Watts DH; for the Pediatric HIVAIDS Cohort Study (PHACS). Safety of tenofovir use during pregnancy: early growth outcomes in HIV-exposed uninfected infants. AIDS. 2012 Feb 29. [Epub ahead of print]
53. Benaboud S, Pruvost A, Coffie PA, Ekouévi DK, Urien S, Arrivé E, Blanche S, Théodoro F, Avit D, Dabis F, Tréluyer JM, Hirt D. Concentrations of tenofovir and emtricitabine in breast milk of HIV-1-infected women in Abidjan, Cote d'Ivoire, in the ANRS 12109 TEmAA Study, Step 2. Antimicrob Agents Chemother. 2011 Mar;55(3):1315-7.
54. Van Rompay KK, Hamilton M, Kearney B, Bischofberger N. Pharmacokinetics of tenofovir in breast milk of lactating rhesus macaques. Antimicrob Agents Chemother 2005 May;49(5):2093-4.
55. Benaboud S, Pruvost A, Coffie PA, Ekouévi DK, Urien S, Arrivé E, Blanche S, Théodoro F, Avit D, Dabis F, Tréluyer JM, Hirt D. Concentrations of tenofovir and emtricitabine in breast milk of HIV-1-infected women in Abidjan, Cote d'Ivoire, in the ANRS 12109 TEmAA Study, Step 2. Antimicrob Agents Chemother. 2011 Mar;55(3):1315-7.
56. Shennan DB, Peaker M. Transport of milk constituents by the mammary gland. Physiol Rev 2000 Jul;80(3):925-51.
57. Kovacs CS, Fuleihan Gel H. Calcium and bone disorders during pregnancy and lactation. Endocrinol Metab Clin North Am 2006 Mar;35(1):21-51.
58. Rillema JA. Effect of prolactin on phosphate transport and incorporation in mouse mammary gland explants. Am J Physiol Endocrinol Metab 2002 Jul;283(1):E132-7.
59. McManaman JL, Neville MC. Mammary physiology and milk secretion. Adv Drug Deliv Rev 2003 Apr 29;55(5):629-41.
60. Loud KJ, Gordon CM. Adolescent bone health. Arch Pediatr Adolesc Med 2006 Oct;160(10):1026-32.
61. Oliveri B, Parisi MS, Zeni S, Mautalen C. Mineral and bone mass changes during pregnancy and lactation. Nutrition 2004 Feb;20(2):235-40.
62. Schiefke I, Fach A, Wiedmann M, Aretin AV, Schenker E, Borte G, et al. Reduced bone mineral density and altered bone turnover markers in patients with non-cirrhotic chronic hepatitis B or C infection. World J Gastroenterol 2005 Mar 28;11(12):1843-7.
63. Kalef-Ezra JA, Merkouropoulos MH, Challa A, Hatzikonstantinou J, Karantanas AH, Tsianos EV. Amount and composition of bone minerals in chronic liver disease. Dig Dis Sci 1996 May;41(5):1008-13.
64. Tsuneoka K, Tameda Y, Takase K, Nakano T. Osteodystrophy in patients with chronic hepatitis and liver cirrhosis. Journal of Gastroenterology 1996;31(5):669-78.
65. Christou H, Connors JM, Ziotopoulou M, Hatzidakis V, Papathanassoglou E, Ringer SA, et al. Cord blood leptin and insulin-like growth factor levels are independent predictors of fetal growth. J Clin Endocrinol Metab 2001 Feb;86(2):935-8.
66. Garnero P, Hausherr E, Chapuy MC, Marcelli C, Grandjean H, Muller C, et al. Markers of bone resorption predict hip fracture in elderly women: the EPIDOS Prospective Study. J Bone Miner Res 1996 Oct;11(10):1531-8.
67. Greenspan SL, Parker RA, Ferguson L, Rosen HN, Maitland-Ramsey L, Karpf DB. Early changes in biochemical markers of bone turnover predict the long-term response to alendronate therapy in representative elderly women: a randomized clinical trial. J Bone Miner Res1998 Sep;13(9):1431-8.
68. Greenspan SL, Rosen HN, Parker RA. Early changes in serum N-telopeptide and C-telopeptide cross-linked collagen type 1 predict long-term response to alendronate therapy in elderly women. J Clin Endocrinol Metab 2000 Oct;85(10):3537-40.
69. Greenspan SL, Resnick NM, Parker RA. Early changes in biochemical markers of bone turnover are associated with long-term changes in bone mineral density in elderly women on alendronate, hormone replacement therapy, or combination therapy: a three-year, double-blind, placebo-controlled, randomized clinical trial. J Clin Endocrinol Metab 2005 May;90(5):2762-7.

70. Glesby MJ. Bone disorders in human immunodeficiency virus infection. Clin Infect Dis2003;37 Suppl 2:S91-5.
71. Kalkwarf HJ, Zemel BS, Gilsanz V, Lappe JM, Horlick M, Oberfield S, et al. The bone mineral density in childhood study: bone mineral content and density according to age, sex, and race. J Clin Endocrinol Metab 2007 Jun;92(6):2087-99.
72. Baim S, Leonard MB, Bianchi ML, Hans DB, Kalkwarf HJ, Langman CB, et al. Official Positions of the International Society for Clinical Densitometry and executive summary of the 2007 ISCD Pediatric Position Development Conference. J Clin Densitom 2008 Jan-Mar;11(1):6-21.
73. Harrison CM, Johnson K, McKechnie E. Osteopenia of prematurity. Act Paediatr 2008 Apr;97(4):407-13
74. Picaud JC, Decullier E, Plan O, Pidoux O, Bin-Dorel S, van Egroo LD, et al. Growth and bone mineralization in preterm infants fed preterm formula or standard term formula after discharge. J Pediatr 2008 Nov;153(5):616-21, 21 e1-2.
75. Akcakus M, Koklu E, Kurtoglu S, Kula M, Koklu SS. The relationship among intrauterine growth, insulinlike growth factor I (IGF-I), IGF-binding protein-3, and bone mineral status in newborn infants. Am J Perinatol 2006 Nov;23(8):473-80.
76. Corbett A, Martinson F, Rezk N, Kashuba A, Jamieson D, Chasela C, et al. Antiretroviral drug concentrations in breast milk and breastfeeding infants. 15th Conference on Retroviruses and Opportunistic Infections; Boston, MA 2008.
77. Schneider S, Peltier A, Gras A, Arendt V, Karasi-Omes C, Mujawamariwa A, et al. Efavirenz in human breast milk, mothers', and newborns' plasma. J Acquir Immune Defic Syndr 2008 Aug 1;48(4):450-4.
78. Zeh C, Weidle P, Nafisa L, editors. Emergence of HIV-1 drug resistance among breastfeeding infants born to HIV-infected mothers taking antiretrovirals for prevention of mother to child transmission of HIV: the Kisumu Breastfeeding Study, Kenya. 15th Conference on Retroviruses and Opportunistic Infections; 2008; Boston, MA.
79. Kent JC, Mitoulas L, Cox DB, Owens RA, Hartmann PE. Breast volume and milk production during extended lactation in women. Exp Physiol 1999 Mar;84(2):435-47.
80. Edginton AN, Schmitt W, Voith B, Willmann S. A mechanistic approach for the scaling of clearance in children. Clin Pharmacokinet 2006;45(7):683-704.

APPENDIX I: MATERNAL P1084s SCHEDULE OF EVALUATIONS

	1077BA or 1077FA Participants Only		1077BP Participants Only	All Participants			
Procedure	Entry[1]	Delivery or Wk 1[2]	Entry/ Wk 1[3]	Wk 6	Wk 26	Wk 74	Early D/C
**CLINICAL EVALUATIONS**							
Informed Consent for Substudy Enrollment	X		X				
Physical Activity History (GPAQ)[4]				X		X	X
Nutrition History/ (24 hour recall)[4]	X			X		X	X
**LABORATORY EVALUATIONS**							
Serum chemistries[5]	X	X	X	X	X	X	X
Urine pregnancy test (before DXA)[6]						X	X
DXA of Spine and Hip[7]		X	X			X	
Plasma storage for TFV[8]		X	X	X	X	X	X
Cord Blood[9] (for TFV level, markers of bone turnover, bone mineral metabolism, vitamin, hormone and inflammatory marker)		X					
Urine Collection[10] (Storage for electrolytes, chemistries, bone turnover markers)	X	X	X	X	X	X	X
Serum Storage[11] (for Chemistries, Markers of Bone Turnover, Bone Mineral Metabolism, Vitamin, Hormone, Inflammatory markers)		8mL	8mL	8mL	8mL	8mL	8mL
Breastmilk Storage[12] (from 1077BF-no additional collection needed)		X	X	X	X	X	
P1084s Maternal Blood Volume (excludes cord blood)		8mL	8mL	8mL	8mL	8mL	8mL
1077 Maternal Blood Volume	21-27mL	15-30mL	15-24mL	21-25mL	24-29mL	24-25mL	24-31mL
Total (P1084s and 1077) Maternal Blood Volume	21-27mL	23-38mL	23-32mL	29-33mL	32-37mL	32-33mL	32-39mL

NOTE: The following maternal procedures/assessments will be completed as part of the main PROMISE protocol: history including fracture history, interval signs and symptoms, physical exam, adherence assessment, smoking status, alcohol intake status, serum creatinine, creatinine clearance. Breast milk from mothers who are breastfeeding will be stored for ARV drug levels.

1. For 1077BA and FA antepartum entry into P1084s should be targeted to occur prior to study drug initiation however women can be enrolled up to the Week 2 visit of 1077 BA or FA, (within 21 days) of entry into 1077BA and FA (prior to labor).
2. Evaluations (excluding DXA, which may be obtained up to 21 days post-partum) are to occur between the Delivery visit and the Week 1 visit (up to Day 14 postpartum).
3. Evaluations (excluding DXA, which may be obtained up to 21 days post-partum) for Entry/Wk 1 visit for women who enter the Postpartum Part of P1084s are to occur at the Week 1 visit (Day 6 – 14 postpartum).
4. To be done at African sites approved as a DXA site.
5. Chemistries (phosphorus and calcium) will be ordered on the sample for chemistries from the parent study, 1077FF or 1077BF (no additional blood required).
6. Pregnancy should be excluded before DXA scans. Ideally, testing should be performed on the same day as the DXA scan but may be done up to and including 14 days prior. Pregnancy test is not required through 21 days postpartum.
7. Only women receiving care at sites with DXA will consent for and undergo DXA. DXAs may be performed on a different day from other evaluations.
8. Stored plasma will be available from the main study for TFV levels.
9. Cord blood collected if reasonably obtainable, e.g. during normal business hours (See Laboratory Section of the MOP and the Lab Processing Chart for details).
10. Urine should preferably be second void of the day. Electrolytes/chemistries on stored urine will include: creatinine, phosphate, calcium, glucose, protein. Measurements on stored urine for bone turnover include d-pyr and NTx (and creatinine).
11. Each 1 ml of blood can be expected to yield 0.5 mL serum or plasma. Listed in priority order: Chemistries (creatinine, phosphorous, calcium); Bone Turnover Markers; CTX; 25-OH Vitamin D; PTH; Inflammatory Cytokines; and Bone Regulatory Cytokines (See Lab Processing Chart for details).
12. Breast milk (from mothers on 1077BA or 1077BP who are breastfeeding), will be stored at Weeks 1, 6, 26 and 74 for mineral composition analysis. See Lab Processing Chart for special processing of the breast milk specimen.

NOTE: Acceptable visit windows for visits are as follows: +/- 1 week for the 6 week visit, and +/- 6 weeks for the 26 and 74 week visits. The Labor and Delivery visit may occur within through the Week 1 visit (up to Day 14 postpartum). Efforts should be made to coordinate mother and infant visits.

APPENDIX II: INFANT P1084s SCHEDULE OF EVALUATIONS

	All Participants				
Procedure	Entry[1]	Wk 10	Wk 26	Wk 74	Early D/C
DXA Scans [2]					
Whole Body (WB)	X (infants enrolled in AP Part only)				
Lumbar Spine (LS)	X		X		
Chemistries[3] (creatinine, calcium & phosphorous testing)	X	X (1mL)	X (0-1mL)	X (0-1mL)	1mL
Urine Collection[4] (Storage for electrolytes, chemistries & bone turnover markers)	X	X	X	X	X
Serum Storage[5] (for Chemistries, Markers of Bone Turnover, Bone Mineral Metabolism, Vitamin, Hormone & Inflammatory markers)	1mL	6-8mL	6-7mL	6-7mL	6 – 7mL
P1084s Infant Blood Volume (mL)	1mL	6-8mL	6-8mL	6-8mL	7-8mL
1077 Infant Blood Volume (mL)	5mL	0 mL	3-6mL	1-5mL	3-5mL
Total Infant Blood Volume (P1084s and 1077)	6mL	6-8mL	9-14mL	7-13mL	10-13mL

Note: The following infant procedures/assessments will be done as part of the main PROMISE protocol: birth and neonatal medical history, interval history, physical (height, weight, head circumference, fontanel status), motor milestones, infant feeding practices assessment. Plasma will be stored from infants for ARV levels. Plasma samples only obtained from infants whose mothers have taken TDF in prior week as part of the main PROMISE protocol. (At least 0.5 mL plasma is required.) No additional blood volume required. Plasma obtained at infant 6 weeks visit will also be available for TFV level.

1. **Antepartum P1084s Entry (1077BA and 1077FA infants):** Evaluations (excluding DXA, which may be obtained up to 21 days post-partum) are to occur between the Birth and Week 1 visit (up to Day 14 of life). For infants participating in 1077BA or 1077FA, serum for chemistries will be obtained at the Week 1 visit as part of the main study.
   Postpartum P1084s Entry (1077BP): Entry Visit (wk 1) for 1077BP infants can be completed through Day 6 – 14 post birth. This Entry Visit should take place at the same time as main PROMISE protocol visit.
2. DXA Scans will be performed at or after Entry Visit for P1084s (up to 21 days of age). Only infants receiving care at sites with DXA will be consented for and undergo DXA. DXAs may be performed on a different day from other evaluations.

3. Chemistries (creatinine, calcium and phosphorous): 1ml whole blood to ensure required minimum of 0.2mL serum will be drawn as part of P1084s at certain visits (see chart below). Some visits will have shared draws with the main studies (1077BF/FF) and a "0mL" in the chart indicates that there is no additional blood needed for P1084s at those visits.

**P1084s Chemistries:** creatinine, calcium & phosphorous	Entry	Wk 10	Wk 26	Wk74	Early D/C
**Participant Subsets:**					
• Infants Co-Enrolled on 1077BF (BA, BP) and on NVP	0mL (drawn on main study)	1mL	0mL (drawn on main study)	0mL (drawn on main study)	1mL
• Infants Co-Enrolled on 1077BF (BA, BP) and NOT on NVP	0mL (drawn on main study)	1mL	0mL (drawn on main study)	1mL	1mL
• Infants Co-Enrolled on 1077FF	0mL (drawn on main study)	1mL	1mL	1mL	1mL

3. Urine collected by urine bag. Electrolytes/chemistries on stored urine will include: creatinine, phosphate, calcium, glucose, protein. Measurements on stored urine for bone turnover include d-pyr and NTx (and creatinine).
4. Each 1mL of blood can be expected to yield 0.5 mL serum or plasma. Listed in priority order: Bone turnover markers; BSAP and osteocalcin; s-CTx. 25OH-Vitamin D; PTH; IGF-1; Inflammatory cytokines (TNF alpha, IL-6, IL-1 beta); and Bone regulatory cytokines (RANKL/OPG) (See Lab Processing Chart for details). If not obtained at entry, should be obtained at 1 week visit. Entry (or 1 week) specimen for stored serum limited to only 0.5 mL serum; for subsequent visit, goal is 6mL blood (about 3ml serum), with prioritization of testing if unable to collect full volume.

NOTE: Acceptable visit windows for infants co-enrolled in 1077BF are as follows: +/- 1 week through cessation of breastfeeding and +/- 4 weeks thereafter. Acceptable visit windows for infants co-enrolled in 1077FF are +/- 2 weeks for week 10 and +/- 4 weeks for weeks 26 and 74. Efforts should be made to coordinate mother and infant visits and visits with the main study.

APPENDIX III: INFORMATION REGARDING RADIATION EXPOSURE FOR IRBS

Mothers: The effective radiation exposure associated with DXA scans of the spine and hip is 0.22 mrem and 0.46 mrem, respectively. Each maternal DXA scan visit involves scans of the spine and hip; with up to three tries for each scan per visit, the maximum radiation exposure per visit is 2.04 mrem. With two DXA visits during the course of the study, the total maximum radiation exposure over the course of the study is 4.08 mrem.

Infants: The maximum numbers of infant scans for the first DXA scan visit involves scans of both the whole body and lumbar spine; with up to three tries for each scan, the maximum radiation exposure is 4.08 mrem. The second infant DXA scan visit involves a scan of the lumbar spine only; with up to three tries for the scan; the maximum radiation exposure is 1.41 mrem, giving a total maximum radiation exposure over the course of the study of 5.49 mrem.

Thus, over the course of the 74 week study, the maximum, cumulative radiation exposure related to DXA will be ≤ 5 mrem for women and ≤ 6 mrem for infants. These doses are comparable to the dose received from naturally occurring sources over a 10 day period at sea level (~ 0.8 mrem/day).

The maximum radiation doses allowed in human research subjects vary by IRB. The guidelines below are based on the Food and Drug Administration's allowable dose limits for radioactive drugs used in research.

Title 21 CFR (Code of Federal Regulations) Part 361.1 (b)(3) “Limit on radiation dose” states: "The amount of radioactive material to be administered shall be such that the subject receives the smallest radiation dose with which it is practical to perform the study without jeopardizing the benefits to be obtained from the study.

(i) Under no circumstances may the radiation dose to an adult research subject from a single study or cumulatively from a number of studies conducted within 1 year be generally recognized as safe if such doses exceed the following:

Whole body, active blood-forming organs, lens of the eye, and gonads:

Single dose	3,000 mRems
Annual and total dose commitment	5,000 mRems

Other organs:

Single dose	5,000 mRems
Annual and total dose commitment	15,000 mRems

(ii) For a research subject under 18 years of age at his last birthday, the radiation dose shall not exceed 10 percent of that set forth in paragraph (b)(3)(i) of this section.

(iii) All radioactive material included in the drug either as essential material or as a significant contaminant or impurity shall be included when determining the total doses and dose commitments. Radiation doses from x-ray procedures that are part of the research study (i.e. would not have occurred but for the study) shall also be included. The possibility of follow-up studies shall be considered for inclusion in the dose calculations.”

APPENDIX IV: SAMPLE INFORMED CONSENT

Informed Consent Form
IMPAACT P1084s
Maternal and Infant Monitoring for Evidence of Toxicity Related to Tenofovir Exposure:
The Bone and Kidney Health Substudy
of the IMPAACT 1077 PROMISE Study
(Promoting Maternal and Infant Survival Everywhere)
Protocol Version 2.0, Dated 11 October 2012

Short Title for the Substudy:
The Bone and Kidney Health Substudy of the 1077 PROMISE Protocol

INTRODUCTION

You and your baby are being asked to take part in this additional research study because:

- You are enrolled in the Antepartum or Postpartum component of the PROMISE Study
- You are infected with human immunodeficiency virus (HIV), the virus that causes AIDS

This substudy is sponsored by the National Institutes of Health (NIH). The doctor in charge of this substudy at this site is: *[insert name of Principal Investigator]*. Before you decide if you want you and your baby to join this additional research study as a pair, we want you to know about the study. We will explain the study to you and you are free to ask questions at any time. We will ask you if you want to join the study along with your baby. If you do want to join with your baby, we will ask you to sign or mark this consent form. You will be offered a copy to take home with you.

WHY IS THIS SUBSTUDY BEING DONE?

The goal of this substudy is to look at the effects on bone health and kidneys of different HIV drugs during pregnancy or while breastfeeding. This substudy will compare the changes in bone health and how the kidney works in women who are taking HIV treatment combinations that include the drug tenofovir to drug combinations that do not include tenofovir. This study will also look at the changes in bone health, how the kidney works and growth (fetal and after birth) in the infants of women taking these drug combinations. A small number of adults (who are not pregnant) and children who take HIV drugs develop problems with their kidneys and with strength of their bones. These problems may be more common when tenofovir is one of the HIV drugs used. Studies about these kidney and bone effects have not yet been done in pregnant and breastfeeding women and their infants. This substudy will help us find out about that information.

WHAT WILL MY BABY AND I HAVE TO DO IF WE ARE IN THIS STUDY?

If you agree for you and your baby to be in this Bone and Kidney Health Substudy, you and your baby will have some more tests added to those done for the main PROMISE Study. We will try to have all study visits and tests needed for this substudy done at the same time you have tests done in the main PROMISE Study. It is expected that each substudy visit will add about *[sites—insert local information on time required for study visits]* to the total time spent in the clinic at each visit. Just as for the main PROMISE Study, you and your baby will have routine medical check-ups at the study clinic. It is important that you and your baby return for all of these study visits. If you do not come for a study visit or if a test result comes back abnormal, the outreach worker will

contact you to find out how you and your baby are doing. If at any time, you or your baby becomes sick you should let the study nurse or doctor know right away. Joining or continuing in this additional research study is voluntary. You can stop the study at any time.

Tests and procedures at the study visits

- Medical history
  We will ask you questions about your health and that of your baby. Some of these questions will be about physical activity and nutrition.

- Blood collection and storage
  Blood will be collected from you as part of the main PROMISE Study. Additional blood will be collected at each visit for this substudy. The total amount of blood taken from you at PROMISE Study visits, including blood for this substudy, will be no more than 41 mLs or about 3 tablespoons *[sites—insert local relevant wording]*.

  Blood will be collected from your baby as part of the main PROMISE Study. At most visits, additional blood will be collected for this substudy. The total amount of blood taken from your baby at PROMISE Study visits, including blood for this substudy, will not be more than 9 mL or about 2 teaspoons *[sites—insert local relevant wording]* for visits up to 10 weeks of age . For visits when your baby is older, the total volume of blood taken at any PROMISE Study visit, including blood for this substudy, will not be more than 16 mL or about 3 teaspoons *[sites—insert local relevant wording]*.

  Blood from you and your baby will be stored during the study for testing the levels of HIV drugs in the blood.

- Urine
  At most visits we will collect urine from you and your baby.

- Special x-ray of bone strength, called a DXA scan *[sites to add if DXA to be done]*
  Within 21 days after you deliver your baby and one more time during the study, we will ask to do a special type of x-ray on you and your baby. This x-ray is done to measure how hard or strong your bones are (called bone density). It is called a DXA scan, which stands for "dual energy x-ray absorptiometry." For the DXA scan, you will lie still on a table for up to 15 minutes while a machine passes over your body and takes a special x-ray of your bones. For your baby's scan, your baby will be held while the scan passes over him/her. The DXA scan does not hurt.

- Cord blood
  Depending on where and when you deliver, we will collect cord blood. Cord blood is collected from the umbilical cord immediately after the birth of the baby, after the cord has been cut. This will not cause any pain to you or your baby.

- Breast milk
  Some of your breast milk collected as part of the PROMISE Study will be stored and used for this substudy.

- Leftover Specimens
  All leftover specimens will be destroyed after study-related testing has been completed. Specimens will not be stored as part of P1084s for future unspecified research.

Summary of the tests that will be done in this substudy:

- Tests of blood and urine to see if you and your baby are losing or gaining bone.
- Tests of hormones, minerals and vitamins that affect bone strength through testing of blood, cord blood, breast milk and urine.
- Blood tests that measure how much the body is reacting to infections.
- Blood test for levels of HIV drugs.
- A special x-ray, called a DXA scan, to measure how strong bones are (this will only be done at some sites).

Some of the basic blood test results will be made available to you and your doctor soon after you and your baby have been seen. At the end of the study, we will send the results of the last DXA scan to your doctor so that your doctor can counsel you about your bone health and the bone health of your baby *[only for sites doing DXA]*. Some of the other special test results on you and your baby will not be available to you.

WHAT IF MY BABY AND I STOP THE SUBSTUDY EARLY?

If you stop or your baby stops the study early before all study visits have been completed, you and your baby will be asked to return to the clinic for a final visit for this substudy that may last about 2 hours. At this final visit you will be asked to provide a urine sample and blood sample (8 mL or about 1 ½ teaspoons *[site to include local language]*). You will be asked to allow your baby to have a urine and blood (about 8 mL or about 1 ½ teaspoons *[site to include local language]*) sample taken at this final visit.

OTHER INFORMATION

The information collected in this bone and kidney health study may be used for other IMPAACT-approved HIV-related research.

HOW MANY WOMEN AND CHILDREN WILL TAKE PART IN THE PROMISE STUDY?

About 875 women and their infants will take part in this Bone and Kidney Health Substudy of the PROMISE Study around the world.

HOW LONG WILL MY BABY AND I BE ON THE PROMISE STUDY?

You and your baby will be followed in the Bone and Kidney Health Substudy for 74 weeks.

WHY MIGHT THE DOCTOR TAKE ME OR MY BABY OFF THIS STUDY EARLY?

The study doctor may need to take you or your baby off the study early without your permission if the study is cancelled or stopped.

The study doctor may also need to take you or your baby off the study if:

- you and your baby are not able to attend the study visits
- you or your baby need a treatment that you may not take while on the study

WHAT ARE THE RISKS OF THE STUDY?

Taking part in this study may involve some risks or discomforts. There is no additional risk for the collection and use of the blood and breast milk samples that will be used for this study. There are no risks to collection of urine, although sometimes it may be uncomfortable for some people. There are no risks to the collection of cord blood.

[Next section to be added for sites doing DXA]

Risk of the DXA Scans

Risk of Radiation:
There is a risk from being near x-rays, also known as radiation. Radiation is energy in the form of waves. Every person is exposed to a small level of radiation from the sun, naturally (called background radiation). High levels of x-rays can lead to cancer. However, the level of x-rays used in a DXA test is much lower than the level that may cause cancer and is about the same as used for a chest x-ray. The study staff has been trained to do the DXA test using the smallest amount of x-ray possible. The level of radiation from the DXA scans in the study is about the same as the "background radiation" (the radiation one gets from the environment) every person gets over 10 days.

Risks Related To Pregnancy
If you become pregnant during the study, you will not be able to have DXA scans because the data could not be interpreted and to minimize unnecessary radiation exposure to the unborn child. Before each DXA scan in this study, you will have a urine pregnancy test. No pregnancy test is needed before DXA scans in the 2 weeks after delivery of your baby, since that is too early after delivery for you to be pregnant again. If you become pregnant, you will not be able to have DXA scans but you will be able to continue with the other tests for the Bone and Kidney Health Substudy.

Possible Risks to Your Privacy

We will make every effort to protect your privacy while you are in this study. Your visits here will take place in private. However, it is possible that others may learn of your participation here and, because of this, may treat you unfairly. There also is a risk to your privacy if someone else taking part in this study knows you.

Other Risks

There may be other risks to taking part in this study which are not known at this time.

ARE THERE BENEFTIS TO ME OR MY BABY TAKING PART IN THIS STUDY?

There may not be a direct benefit to you and your baby. Information learned from the PROMISE Study may help us understand ways to avoid bone and kidney problems during pregnancy or breastfeeding in HIV-infected mothers. You may get some satisfaction from knowing that you

and your baby participated in this study. Having a DXA scan may provide important information for your doctor about your bone health and the bone health of your baby.

WHAT OTHER CHOICES DO MY BABY AND I HAVE BESIDES THIS SUBSTUDY?

Joining or continuing in this substudy is voluntary. If you do not want to be or do not want your baby to join this study and have the additional tests, you and your baby can still be in the main PROMISE Study. Please talk to your doctor about these and other choices available to you and your baby. Your doctor will explain the risks and benefits of these choices. You and your baby will continue to receive regular care whether or not you take part in this substudy.

WHAT ABOUT CONFIDENTIALITY?

Every effort will be made to keep your personal information and that of your baby confidential. This personal information may be disclosed if required by law. Your records will be identified by a study code. Any publication of this study will not use you or your baby's name or identify you or your baby personally.

The outreach workers may contact you so we need to know the best way to reach you (such as home visit or phone call). Your records may be reviewed by the *[insert name of site]* IRB or ethics committee, the US Food and Drug Administration (FDA), the study sponsor (the US National Institutes of Health) or its agents, the US Office of Human Research Protections, local regulatory authorities, study staff, study monitors, and the drug companies supporting this study.

WHAT ARE THE COSTS TO ME?

There are no costs to you for the additional tests and DXA scans that will be done as part of this substudy.

WILL I RECEIVE ANY PAYMENT?

Reimbursement for your time and travel are already covered through your participation in the main PROMISE Study. You may be offered additional reimbursement for the additional time spent in participating in this substudy.

WHAT HAPPENS IF EITHER MY BABY OR I ARE INJURED?

If you are or your baby is injured as a result of being in this study, you will be given immediate treatment for your injuries *[sites: add local information regarding treatment for injury]*. There is no program for compensation either through this study or the sponsor of the study, the National Institutes of Health (NIH). *[sites: do not delete the statement that the sponsor will not provide compensation for injury]*. You will not be giving up any of your legal rights by signing this consent form.

WHAT ARE OUR RIGHTS AS RESEARCH PARTICIPANTS?

Taking part in this substudy is voluntary. You may choose not to participate in this substudy or leave this substudy at any time. If you decide not to participate or to leave the study early, you and your baby will not be penalized or lose any benefits to which you would otherwise have access outside of the study. We will tell you about new information from this or other studies that may affect you and your baby's health, welfare or willingness to stay in this study. If you want to

be informed about the results of this study, the study staff will contact you when these are available. *[sites: include local information about how participants can find out about study results if applicable]*. We will tell you of any new findings that may affect your desire to continue to participate in the study.

WHAT DO I DO IF I HAVE QUESTIONS OR PROBLEMS?

For questions about this study or a research-related injury, contact:

- *[Insert name of the investigator or other study staff]*
- *[Insert telephone number of above]*

For questions about your or your baby's rights as a research subject, contact:

- *[name or title of person on the Institutional Review Board (IRB) or other organization appropriate for the site]*
- *[telephone number of above]*

SIGNATURE PAGE

If you have read this consent form (or had it explained to you), all your questions have been answered and you agree for you and your infant to take part in this study, please sign your name below.

______________________________	______________________________
Participant's Name (print)	Participant's Signature and Date
______________________________	______________________________
Infant Father's Name (print)<br>(If reasonably available)	Father's Signature and Date<br>(If reasonably available)
______________________________	______________________________
Study Staff Conducting<br>Consent Discussion (print)	Study Staff Signature and Date
______________________________	______________________________
Witness Name (print)<br>(As appropriate)	Witness Signature and Date
